# Coding principles and mechanisms of serotonergic transmission modes

**DOI:** 10.1038/s41380-025-02930-4

**Published:** 2025-02-22

**Authors:** Yajun Zhang, Peng Zhang, Mimi Shin, Yuanyu Chang, Stephen B. G. Abbott, B. Jill Venton, J. Julius Zhu

**Affiliations:** 1 Departments of Pharmacology, University of Virginia School of Medicine, Charlottesville, VA 22903 USA; 2 Departments of Chemistry, University of Virginia, Charlottesville, VA 22904 USA; 3 School of Pharmaceutical Sciences, Wenzhou Medical University, Wenzhou, 325035 China

**Keywords:** Neuroscience, Diseases

## Abstract

Serotonin-mediated intercellular communication has been implicated in myriad human behaviors and diseases, yet how serotonin communicates and how the communication is regulated remain unclear due to limitations of available monitoring tools. Here, we report a method multiplexing genetically encoded sensor-based imaging and fast-scan cyclic voltammetry, enabling simultaneous recordings of synaptic, perisynaptic, proximate and distal extrasynaptic serotonergic transmission. Employing this method alongside a genetically encoded sensor-based image analysis program (GESIAP), we discovered that heterogeneous firing patterns of serotonergic neurons create various transmission modes in the mouse raphe nucleus and amygdala, encoding information of firing pulse frequency, number, and synchrony using neurotransmitter quantity, releasing synapse count, and synaptic and/or volume transmission. During tonic and low-frequency phasic activities, serotonin is confined within synaptic clefts due to efficient retrieval by perisynaptic transporters, mediating synaptic transmission modes. Conversely, during high-frequency, especially synchronized phasic activities, or when transporter inhibition, serotonin may surpass transporter capacity, and escape synaptic clefts through 1‒3 outlet channels, leading to volume transmission modes. Our results elucidate a mechanism of how channeled synaptic enclosures, synaptic properties, and transporters collaborate to define the coding principles of activity pattern-dependent serotonergic transmission modes.

## Introduction

Serotonergic transmission is involved in a wide array of human behaviors and diseases [1–3]. The sheer number of brain functions influenced by serotonin, encompassing cognition, mood, social interaction, sexual behavior, feeding behavior, motor behavior, reward, motivation, sleep-wake cycle, and thermoregulation, is closely tied to the diverse firing patterns exhibited by serotonergic neurons [4–9]. Despite this connection, for a very long time, it has remained uncertain how these heterogeneous firing patterns might influence serotonin release and signaling, subsequently impacting associated behaviors and diseases [3, 10]. Decades of research, primarily reliant on low-spatial-resolution voltammetry (and microdialysis) experiments that typically detect only extracellular signals [11], postulates serotonin as a volume transmitter that influences many neighboring cells, largely irrespective of firing patterns [12, 13]. However, an electrophysiology study, which, despite relying on indirect calculations and simulation assumptions, estimated remarkably restrained serotonergic transmission [14]. Moreover, selective serotonin reuptake inhibitors (**SSRIs**) are commonly prescribed treatments for depression, but their effects on serotonin reuptake and release are poorly understood [15–17]. Voltammetric studies on SSRIs have fostered a tacitly accepted, nevertheless pharmacologically counterintuitive notion that SSRIs enhance serotonin release [18–21]. Thus, there is a critical need for a reliable, high-resolution method that enables thorough investigations into essential questions surrounding serotonin, its signaling, and functions. Such a method is crucial for our understanding of related behaviors and the development of treatments for associated disorders [10, 22].

In this study, we developed a multiplexed genetically encoded sensor-based imaging and fast-scan cyclic voltammetry (**FSCV**) method to achieve the first simultaneous scrutiny of synaptic, perisynaptic, proximate and distal extrasynaptic transmission. Nanoscopic visualization and transmission property analysis with genetically-encoded sensor-based image analysis program (**GESIAP**) revealed the modes and regulation of serotonergic transmission. Our research in the mouse raphe nucleus and amygdala unveiled that different firing patterns in serotonergic neurons lead to various transmission modes. These modes encode the frequency, number, and synchrony of firing pulses with neurotransmitter quantity, releasing synapse count, and the type of transmission—synaptic or volume. During both (continuous) tonic and low-frequency (burst-like) phasic activities, efficient retrieval by perisynaptic transporters confines serotonin within synaptic clefts, resulting in synaptic transmission. However, during high-frequency or synchronized activities, or when transporters are inhibited, excess serotonin surpasses transporter capacity, diffusing into synaptic clefts through one, sometimes two, and occasionally three outlet channels, initiating volume-based transmission modes. Our findings provide insights into how synaptic structures, properties, and transporters collaborate to regulate serotonergic transmission based on specific neuronal activity patterns.

## Results

### Multiplexed GRAB_5HT_-based imaging and FSCV_5HT_ recordings

**Fig. 1:**
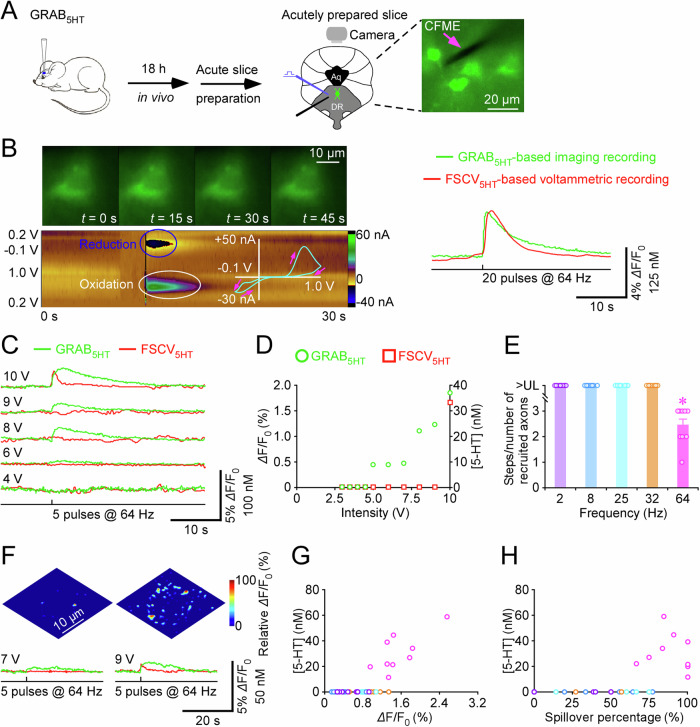
Simultaneous GRAB_5HT_- and FSCV_5HT_-based recordings of serotonergic transmission. **A** Schematic of experimental design illustrating multiplexed imaging and voltammetry conducted in a mouse dorsal raphe nucleus (**DR**) slice preparation. The image on the right depicts the placement of a carbon fiber microelectrode (**CFME**), highlighted by a pink arrow, in proximity to GRAB_5HT_ expressing neurons marked by an orange arrow. Note the distance between CFMEs and imaged expressing neurons in paired recordings, ranging from 12 to 48 µm (26.6 ± 5.8 µm; *n* = 9 pairs from 9 animals). **B** Left, snapshots of fluorescence *Δ*F/F_0_ responses (upper) and a color plot of cyclic voltammogram (lower) of electrically evoked serotonin release at a GRAB_5HT_ expressing raphe neuron. Right, simultaneous GRAB_5HT_-based imaging and FSCV_5HT_-based voltammetric recording traces. **C** GRAB_5HT_ and FSCV_5HT_ recording traces capturing serotonin release in response to stimulus trains composed of 5 pulses at 64 Hz at varied intensities. Note the stepwise increase in GRAB_5HT_ fluorescence *Δ*F/F_0_ responses, suggesting the recruitment of more axons with elevated stimulus intensity. **D** Maximal fluorescence *Δ*F/F_0_ responses and serotonin concentrations measured with GRAB_5HT_ (green) and FSCV_5HT_ (red) in response to the stimulus trains. **E** Summary of GRAB_5HT_ response steps (presumably equivalent to the number of recruited axons) required to trigger FSCV_5HT_ signals under various stimulation parameters (20 pulses at 2 Hz: >upper limit, *n* = 9 neurons from 5 animals; 5 pulses at 8 Hz: >upper limit, *n* = 7 neurons from 4 animals; 5 pulses at 25 Hz: >upper limit, *n* = 8 neurons from 5 animals; 5 pulses at 32 Hz: >upper limit, *n* = 7 neurons from 4 animals; 5 pulses at 64 Hz: 2.44 ± 0.24, *n* = 9 neurons from 5 animals). The step required for 5 pulses at 64 Hz (*p* < 0.001, *U* = 0.00) were significantly smaller than those for 20 pulses at 2 Hz. Asterisks indicate *p* < 0.05 (Rank Sum non-parametric tests). **F** Heatmaps of electrically evoked GRAB_5HT_ fluorescence *Δ*F/F_0_ responses, and GRAB_5HT_ and FSCV_5HT_ recording traces in response to 5 pulses 64 Hz stimuli delivered at voltages of 7 and 9 V. Scale bars applied to all in **F**. **G** Plot of voltammetric FSCV_5HT_ responses against fluorescence *Δ*F/F_0_ responses in response to 20 pulses delivered at 2 Hz, 5 pulses at 8, 25, 32 and 64 Hz. Note the occurrence of voltammetric FSCV_5HT_ responses corresponding to a minimum of 0.60% GRAB_5HT_ fluorescence *Δ*F/F_0_. **H** Plot of voltammetric FSCV_5HT_ responses against percentages of individual releasing synapses showing an expanded serotonin diffusion profile in response to 20 pulses delivered at 2 Hz, 5 pulses at 8, 25, 32, and 64 Hz. Note the occurrence of voltammetric FSCV_5HT_ responses corresponding to a minimum of 32.86% individual release sites exhibiting the expanded serotonin diffusion profile.

To simultaneously monitor synaptic, perisynaptic, and proximate and distal extrasynaptic serotonergic transmission, we multiplexed genetically encoded sensor-based functional imaging and FSCV in a mouse dorsal raphe nucleus ex vivo preparation (Fig. 1A). Specifically, we made Sindbis viral expression of serotonin sensors in the raphe nucleus in vivo and ~18 h after the expression, prepared acute brain raphe slices. Then, we used a local stimulating electrode to activate serotonergic fibers, and simultaneously recorded fluorescence responses in sensor expressing neurons with a Hamamatsu ORCA FLASH4.0 camera and voltammetric responses with a Nafion-coated carbon-fiber microelectrode placed next to sensor expressing neurons (Fig. 1B). Using Nafion-coated carbon-fiber microelectrodes with a serotonin-specific Jackson FSCV voltage waveform permitted serotonin-specific fast-scan cyclic voltammetry (**FSCV**_**5HT**_) detection (Fig. S1; cf. [23, 24]). To selectively image serotonergic fluorescence responses, we first tested two newly developed genetically encoded serotonin sensors, GRAB_5HT_ [25] and iSeroSnFR [26], in cultured hippocampal slices (Fig. S2A), a preparation routinely employed for rapid sensor testing [25, 27, 28]. While a brief 10-ms puff of serotonin evoked fluorescence responses in both GRAB_5HT_ and iSeroSnFR expressing neurons, more robust *Δ*F/F_0_ responses were recorded in GRAB_5HT_ than iSeroSnFR expressing neurons, particularly when a physiological concentration of serotonin was applied (Fig. S2B, C). Thus, GRAB_5HT_ was chosen for all further experiments. To confirm the specificity of *Δ*F/F_0_ responses detected by GRAB_5HT_, which is engineered based on the serotonin 2C receptor [25], we bath applied RS102221, a selective antagonist of the receptor [29], in an ex vivo preparation of the mouse dorsal raphe nucleus, a brain area known for prominent serotonin signals [3]. Bath application of RS102221 abolished the fluorescence responses evoked by a train of 20 electric pluses delivered at 64 Hz, but not voltammetric responses at GRAB_5HT_ expressing neurons (Fig. S2D–H), verifying the specificity of GRAB_5HT_ fluorescence responses.

Serotonergic neurons display a spectrum of firing patterns intricately linked to various behaviors, yet uncovering the precise impact of these firing activities on serotonin release and signaling remains a persistent challenge. In the mouse dorsal raphe nucleus ex vivo preparation, we applied electric stimulations that simulated low-frequency tonic firing at ~2 Hz, short burst firing at ~8, ~25, ~32, and ~64 Hz (Fig. 1C–E), which are associated with behaviors related to wakefulness or alertness, reward expectations, sensory stimuli or natural rewards, unexpected rewards, and sexual or social rewards, respectively [4–9]. We found that only high frequency, high intensity stimuli induced both GRAB_5HT_ fluorescence and FSCV_5HT_ voltammetric responses (Fig. 1C–E). To examine whether the responses depend on the recruitment of more presynaptic axons as electric stimulation intensity increases, we applied a minimal stimulation protocol. Figure 1C, D showed that low-intensity stimuli (3.0, 3.5, 4.0, and 4.5 V) of 5 pulses at 64 Hz evoked neither GRAB_5HT_ fluorescence nor FSCV_5HT_ voltammetric responses. Higher intensity stimuli (5.0, 6.0, and 7.0 V) elicited small, consistent GRAB_5HT_ fluorescence responses, but no FSCV_5HT_ responses. Even higher intensity stimuli (8.0 and 9.0 V) doubled the size of GRAB_5HT_ fluorescence responses, still without FSCV_5HT_ responses. Only the highest intensity stimuli (10.0 V) elicited both tripled-sized GRAB_5HT_ fluorescence responses and FSCV_5HT_ voltammetric responses. These findings suggest that the activation of single serotonergic axons usually results in constrained serotonergic transmission. However, the synchronized activation of multiple serotonergic axons or neuronal pools, as observed in emotional behaviors [30], leads to serotonin dispersion beyond individual GRAB_5HT_ expressing neurons, as detected by FSCV_5HT_, indicating volume transmission. Additionally, lower frequency stimuli, including 5-pulse stimuli at 8, 25, and 32 Hz, along with 20-pulse stimuli at 2 Hz, consistently elicited GRAB_5HT_ fluorescence responses without observable FSCV_5HT_ voltammetric responses across the entire examined intensity range (Fig. 1E), suggesting a restricted serotonergic transmission.

We conducted further quantitative analysis of GRAB_5HT_ fluorescence responses using a genetically encoded sensor-based image analysis program (**GESIAP**) [31], enabling visualization of serotonin diffusion at individual releasing synapses (Fig. 1F). The analysis revealed that instances of low GRAB_5HT_ fluorescence responses (<~0.60% *Δ*F/F_0_) or low percentages of individual releasing synapses exhibiting an expanded serotonin diffusion profile (<~33%) were seldom accompanied by FSCV_5HT_ voltammetric responses (Fig. 1G–H). Only heightened activity could result in enhanced serotonin release and expanded serotonin diffusion, and evident volume transmission (Fig. 1G–H).

### Transporters control serotonin reuptake but not release

**Fig. 2:**
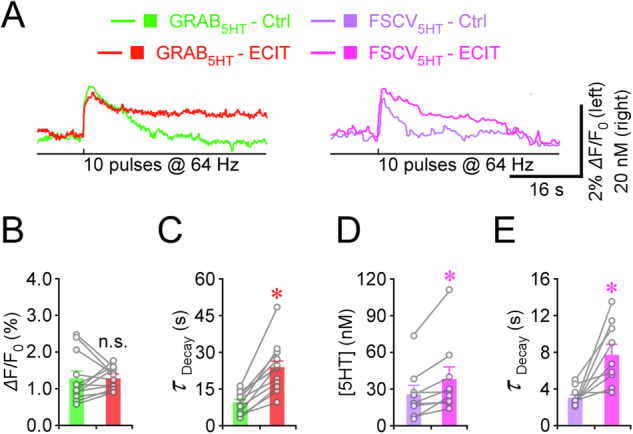
Serotonin transporter inhibition enhances serotonin signals. **A** Simultaneous GRAB_5HT_ and FSCV_5HT_ recordings of evoked voltammetry and fluorescence *Δ*F/F_0_ responses before (green and red) and after (purple and pink) the bath application of 10 µm escitalopram (**ECIT**). **B–C** Peak amplitudes (Ctrl: 1.26 ± 0.19%; ECIT: 1.27 ± 0.08%, *Z* = 0.24, *p* = 0.85, *n* = 12 neurons from 6 animals) and decay time constants (Ctrl: 9.74 ± 1.25 s; ECIT: 23.84 ± 2.88 s, *Z* = 3.06, *p* < 0.001, *n* = 12 neurons from 6 animals) of GRAB_5HT_ fluorescence *Δ*F/F_0_ responses before (green) and after (red) the bath application of ECIT. **D–E** Peak amplitudes (Ctrl: 25.67 ± 6.73 nM; ECIT: 38.22 ± 10.00 nM, *Z* = 2.66, *p* < 0.001, *n* = 9 neurons from 6 animals) and decay time constant (Ctrl: 3.09 ± 0.34 s; ECIT: 7.76 ± 1.14 s, *Z* = 2.66, *p* = 0.004, *n* = 9 neurons from 6 animals) of FSCV_5HT_ voltammetric responses before (purple) and after (pink) the bath application of ECIT. Asterisks indicate *p* < 0.05 (Wilcoxon tests).

To determine whether serotonin transporters control serotonin dispersion beyond individual GRAB_5HT_ expressing neurons, we examined the impact of escitalopram, a potent selective serotonin transporter inhibitor [18, 32], on simultaneously recorded GRAB_5HT_ fluorescence and FSCV_5HT_ voltammetric responses in the mouse dorsal raphe nucleus ex vivo preparation. Bath application of escitalopram prolonged the time course of both GRAB_5HT_ fluorescence and FSCV_5HT_ voltammetric responses evoked a train of 10-pulse stimuli delivered at 64 Hz, although it enhanced the amplitude of only FSCV_5HT_ voltammetric responses but not GRAB_5HT_ fluorescence responses (Fig. 2A–E). The results suggest that escitalopram enhances extracellular serotonergic transmission, consistent with previous FSCV_5HT_-based voltammetric studies [18–21]. However, our GRAB_5HT_-based imaging suggests an alternative mechanism that is mediated by inhibiting reuptaking the transmitter without increasing its release.

**Fig. 3:**
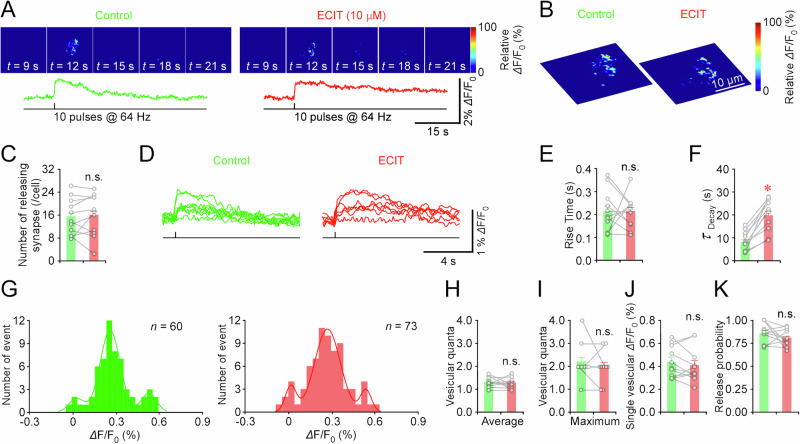
Transporter inhibition prolongs serotonin signals without altering release properties. **A** Heatmaps of electrically evoked fluorescence *Δ*F/F_0_ responses before (left) and after (right) the bath application of 10 µm escitalopram (**ECIT**). **B** 3D spatiotemporal profiling of electrically evoked fluorescence *Δ*F/F_0_ responses before (left) and after (right) the bath application of ECIT. Scale bars applied to all in **A**, **B**. **C** Releasing synapse counts before (green) and after (red) the bath application of ECIT (Ctrl: 15.45 ± 1.86; ECIT: 16 ± 2.10; *Z* = 0.36, *p* = 0.76, *n* = 11 neurons from 5 animals). **D** Ten *Δ*F/F_0_ responses evoked by single pulse stimuli at isolated releasing synapses before (green) and after (red) the bath application of ECIT. **E–F** 10–90% rise times (Ctrl: 0.21 ± 0.03 s; ECIT: 0.21 ± 0.02 s; *Z* = −0.36, *p* = 0.76; *n* = 11 neurons from 5 animals) and decay time constants (Ctrl: 8.22 ± 1.18; ECIT: 19.68 ± 2.04; *Z* = 2.93, *p* < 0.001; *n* = 11 neurons from 5 animals) of *Δ*F/F_0_ responses before (green) and after (red) the bath application of ECIT. **G** Amplitude histograms of *Δ*F/F_0_ responses before (green) and after (red) the bath application of ECIT. **H–K** Average (Ctrl: 1.34 ± 0.06; ECIT: 1.28 ± 0.07; *Z* = −0.46, *p* = 0.69, *n* = 11 neurons from 5 animals) and maximal (Ctrl: 2.18 ± 0.22; ECIT: 2 ± 0.19; *Z* = −0.46, *p* = 0.69, *n* = 11 neurons from 5 animals) vesicular quanta, quantal sizes (Ctrl: 0.44 ± 0.04%; ECIT: 0.41 ± 0.04%; *Z* = −0.89, *p* = 0.41, *n* = 11 neurons from 5 animals), and release probabilities (Ctrl: 86.32 ± 3.4%; ECIT: 80.69 ± 2.72%; *Z* = −1.33, *p* = 0.21, *n* = 11 neurons from 5 animals). Asterisks indicate *p* < 0.05 (Wilcoxon tests).

To verify the findings, we directly measured the effect of escitalopram on the release properties of serotonergic transmission using the genetically encoded sensor-based image analysis program (**GESIAP**) [31]. Bath application of escitalopram lengthened the decay time course of evoked fluorescence GRAB_5HT_ responses without altering the rise time, and it did not change the number of releasing synapses at GRAB_5HT_ expressing neurons that were visualized with GESIAP (Fig. 3A–C; Movie S1). Application of trains of electric pulses at 0.1 Hz evoked *Δ*F/F_0_ responses at single releasing synapses of GRAB_5HT_ expressing neurons that appeared as stochastic failures and release events (Fig. 3D). Bath application of tetrodotoxin (**TTX**), which blocks action potential-dependent synaptic transmission [33], resulted in elimination of the quantal release events (Fig. S3), confirming the synaptic origin of the signals (cf. [31, 34]). Moreover, the bath application of TTX revealed the low occurrence of TTX-insensitive quantal events (0 events per 5–10 min, *n* = 6 neurons from 5 animals), suggesting a high dependence of transmitter release on action potentials. These evoked fluorescence responses were fit with a double-exponential synaptic function using MATLAB algorithms in GESIAP, providing estimates of key parameters such as rise time, decay time constant, and amplitude [31, 35]. Escitalopram did not alter the rise time of quantal release events but did enhance the decay time constant of quantal responses (Fig. 3E, F). Plotting *Δ*F/F_0_ amplitude histograms, an analysis method adapted from the classic quantal analysis approach [36], revealed multiple, nearly equally spaced peaks, and analysis yielded the same number of released vesicles (average ~1.3 vesicular quanta and up to 3–4 vesicular quanta per stimulus) before or after bath application of escitalopram (Fig. 3G–I). In addition, the analysis enabled direct readout of the same vesicular quantal size of ~0.45% *Δ*F/F_0_ for serotonin and the same release probability *P*_r_ of ~0.80 (release success rate over multiple trials) at single releasing synapses before and after bath application of escitalopram (Fig. 3J–K). These results confirm that escitalopram prolongs serotonergic signals by inhibiting reuptake without affecting serotonin release.

**Fig. 4:**
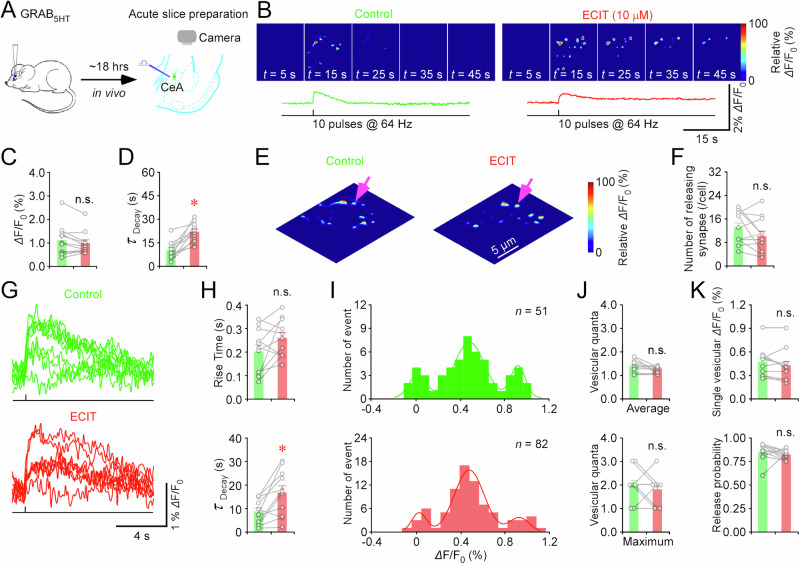
Transporter inhibition prolongs amygdalar serotonin signals without affecting release. **A** Schematic of experiment design in a mouse amygdalar slice preparation. CeA: the central nucleus of the amygdala. **B** Heatmaps of electrically evoked fluorescence *Δ*F/F_0_ responses before (left) and after (right) the bath application of 10 µm escitalopram (**ECIT**) in the amygdala. **C–D** Peak amplitudes (Ctrl: 1.10 ± 0.19%; ECIT: 0.98 ± 0.14%, *Z* = −1.41, *p* = 0.18, *n* = 12 neurons from 6 animals) and decay time constants (Ctrl: 10.20 ± 1.59 s; ECIT: 21.97 ± 1.74 s, *Z* = 3.06, *p* < 0.001, *n* = 12 neurons from 6 animals) of GRAB_5HT_ fluorescence *Δ*F/F_0_ responses before (green) and after (red) the bath application of ECIT. **E** 3D spatiotemporal profiling of electrically evoked fluorescence *Δ*F/F_0_ responses before (left) and after (right) the bath application of ECIT. Scale bars applied to all in **B** and **E**. **F** Releasing synapse counts before (green) and after (red) the bath application of ECIT (Ctrl: 13.09 ± 1.69; ECIT: 10.64 ± 1.76; *Z* = −1.17, *p* = 0.28, *n* = 11 neurons from 6 animals). **G** Ten *Δ*F/F_0_ responses evoked by single pulse stimuli at isolated releasing synapses before (green) and after (red) the bath application of ECIT. **H** 10–90% rise times (Ctrl: 0.21 ± 0.03 s; ECIT: 0.26 ± 0.02 s; *Z* = 1.74, *p* = 0.08; *n* = 11 neurons from 6 animals) and decay time constants (Ctrl: 8.61 ± 1.56; ECIT: 16.60 ± 2.83; *Z* = 2.58, *p* = 0.01; *n* = 11 neurons from 6 animals) of *Δ*F/F_0_ responses before (green) and after (red) the bath application of ECIT. **I** Amplitude histograms of *Δ*F/F_0_ responses before (green) and after (red) the bath application of ECIT. **J–K** Average (Ctrl: 1.37 ± 0.08; ECIT: 1.18 ± 0.05; *Z* = −1.72, *p* = 0.10, *n* = 11 neurons from 6 animals) and maximal (Ctrl: 2 ± 0.19; ECIT: 1.82 ± 0.18; *Z* = −0.82, *p* = 0.56, *n* = 11 neurons from 6 animals) vesicular quanta, quantal sizes (Ctrl: 0.47 ± 0.05%; ECIT: 0.43 ± 0.06%; *Z* = −1.78, *p* = 0.08, *n* = 11 neurons from 6 animals), and release probabilities (Ctrl: 84.59 ± 2.8%; ECIT: 81.70 ± 1.49%; *Z* = −1.17, *p* = 0.27, *n* = 11 neurons from 6 animals). Asterisks indicate *p* < 0.05 (Wilcoxon tests).

To independently corroborate our findings in a different brain region, we investigated the effect of escitalopram on serotonin diffusion in an ex vivo amygdalar preparation, another brain area with prominent serotonin signals [3]. When escitalopram was introduced into the bath solution, we observed a prolonged decay time course of evoked fluorescence responses at GRAB_5HT_ expressing amygdalar neurons, while the response amplitude remained unchanged (Fig. 4A–D). We did not observe any significant alterations in the number of releasing synapses at GRAB_5HT_ expressing amygdalar neurons (Fig. 4E, F). Moreover, while escitalopram did not affect the rise time of quantal release events, it notably extended the decay time constant of these responses (Fig. 4G, H). Quantal analysis revealed consistent numbers of released vesicles, averaging ~1.3 vesicular quanta, occasionally up to 2–3 vesicular quanta per stimulus before and after escitalopram application (Fig. 4I, J). Furthermore, this analysis showed the same vesicular quantal size of ~0.45% *Δ*F/F_0_ for serotonin and the release probability at single releasing synapses remained unchanged, maintaining a value of ~0.80 at single releasing synapses before and after escitalopram application (Fig. 4K). Together, these results suggest that the escitalopram-induced inhibition of transporters prolongs serotonergic signals without directly affecting the release of serotonin itself.

To further validate our observations using an alternative pharmacological inhibitor, we employed paroxetine, a more potent serotonin transporter inhibitor [18], in the mouse ex vivo amygdalar preparation. Paroxetine prolonged the decay time course of evoked fluorescence GRAB_5HT_ responses without altering the response magnitude (Fig. S4A–D). Moreover, the quantity of releasing synapses in these neurons remained unchanged (Fig. S4E, F). Once again, paroxetine did not impact the rise time of quantal release events but significantly prolonged their decay time constant (Fig. S4G, H). Quantitative analysis consistently indicated similar numbers of released vesicles, averaging ~1.2 vesicular quanta and occasionally up to 2–3 vesicular quanta per stimulus before and after paroxetine application (Fig. S4I, J). Additionally, this analysis reaffirmed a consistent vesicular quantal size of ~0.40% *Δ*F/F_0_ for serotonin, maintaining an unaltered release probability of ~0.80 at single releasing synapses before and after paroxetine application (Fig. S4I, J). Collectively, these findings consistently support the notion that transporter inhibition extends serotonergic signals without directly influencing serotonin release.

### Somatic and dendritic releasing synapses share the same properties

**Fig. 5:**
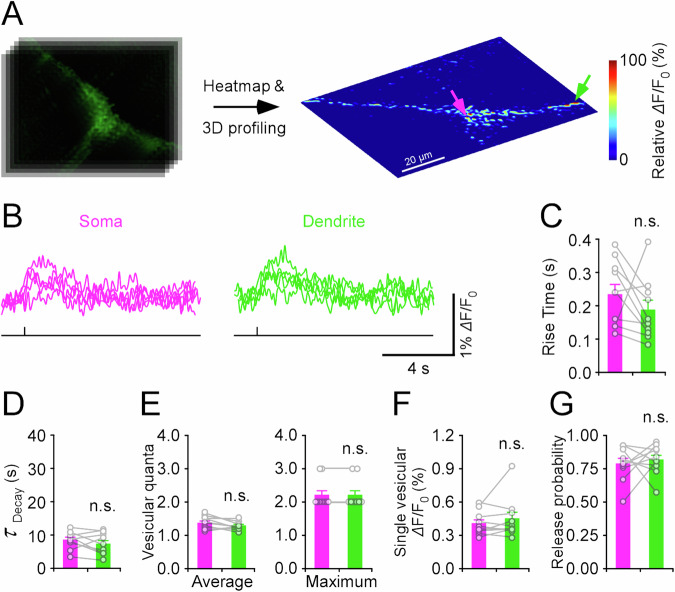
Somatic and dendritic releasing synapses share the same properties. **A** Snapshots and 3D spatiotemporal profiling of electrically evoked fluorescence *Δ*F/F_0_ responses in the CeA. Note the isolated releasing synapses on the soma and dendrite indicated by pink and green arrows, respectively. **B** *Δ*F/F_0_ responses evoked by single pulse stimuli at isolated releasing synapses on the soma (pink) and dendrite (green). **C–D** 10–90% rise times (Soma: 0.24 ± 0.03 s; Dendrite: 0.19 ± 0.03 s; *U* = 33.50, *p* = 0.23) and decay time constants (Soma: 8.35 ± 0.81 s; Dendrite: 7.11 ± 1.00 s; *U* = 40.00, *p* = 0.47) of *Δ*F/F_0_ responses. **E–G** Average (Soma: 1.37 ± 0.06; Dendrite: 1.27 ± 0.04; *U* = 33.00, *p* = 0.21) and maximal (Soma: 2.20 ± 0.13; Dendrite: 2.20 ± 0.13; *U* = 50.00, *p* = 0.96) vesicular quanta, quantal sizes (Soma: 0.40 ± 0.03%; Dendrite: 0.44 ± 0.06%; *U* = 56.50, *p* = 0.65), and release probabilities (Soma: 78.00 ± 4.00%; Dendrite: 81.40 ± 3.47%; *U* = 59.5, *p* = 0.50) on the soma (green) and dendrite (cyan). Note n.s. indicates no statistical difference (*p* > 0.05; *n* = 10 neurons from 5 animals; Rank Sum non-parametric tests).

At times, 1–2 dendrites were observed in the same focal plane as the soma of GRAB_5HT_ expressing neurons in the amygdalar preparation, enabling simultaneous imaging of GRAB_5HT_ fluorescence responses at single releasing synapses on both the soma and dendrites of GRAB_5HT_ expressing neurons (Fig. 5A). The evoked quantal events at these locations exhibited the identical rise times and decay time constants (Fig. 5B–D). Synaptic properties at somatic and dendritic synapses were consistent, with an average of ~1.2 vesicular quanta per stimulus and occasional events of 2–3 vesicular quanta (Fig. 5E). The vesicular quantal size was ~0.40–0.45% *Δ*F/F_0_ for serotonin, with a release probability *P*_r_ of ~0.80 at both somatic and dendritic synapses (Fig. 5F, G). These results suggest that the basic properties of serotonergic synapses are conserved between the soma and dendrites, reinforcing the idea that these properties are consistent across various brain areas and animal species [31, 34].

Given the consistent vesicular quantal size across all synapses, we sought to estimate the amount of serotonin released during quantal events, which remains unclear due to limitations in current detection methods, such as electrochemistry, microdialysis, and fluorescent sensor-based photometry [11, 37]. To address this, we calibrated the serotonin concentration-fluorescence response curve for GRAB_5HT_, which was fitted with a sigmoidal function (Fig. S5). Using this function, we converted the vesicular quantal size of ~0.45% *Δ*F/F_0_ to an estimated ~100 nM of serotonin at individual releasing synapses on both the soma and dendrites.

### Transporters gate serotonin egress at synapses

**Fig. 6:**
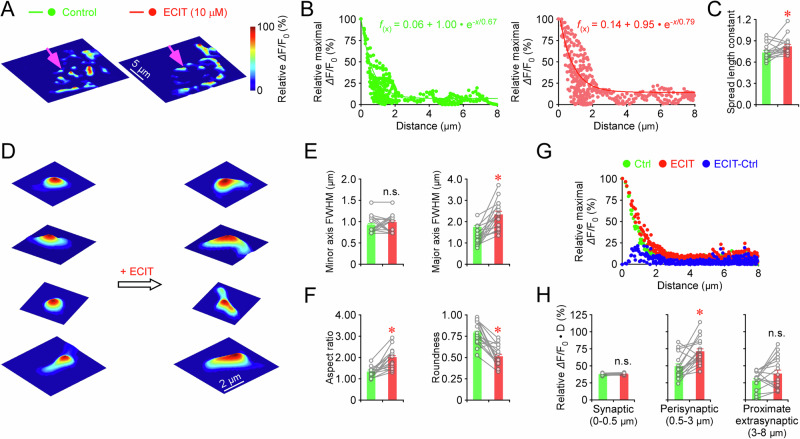
Transporter inhibition drives extrasynaptic diffusion of serotonin. **A** 3D spatiotemporal profiling of electrically evoked fluorescence *Δ*F/F_0_ responses before (left) and after (right) the bath application of 10 µm escitalopram (**ECIT**). **B** Pixel-wise maximal *Δ*F/F_0_ plots before (left) and after (right) the bath application of ECIT at the same isolated release site indicated by the pink arrows in **A**. **C** Averaged spatial spread length constants (Ctrl: 0.72 ± 0.03 µm; ECIT: 0.84 ± 0.04 µm, *Z* = 2.67, *p* = 0.007, *n* = 17 releasing synapses from 5 animals). **D** 3D profiling of *Δ*F/F_0_ responses at isolated releasing synapses before and after the bath application of ECIT. Note serotonin leaking out of synapses via 1 − 3 outlets after the bath application of ECIT (1.53 ± 0.17; *n* = 17 releasing synapses from 5 animals). Scale bar applied to all in **D**. **E** Minor (Ctrl: 0.93 ± 0.05 µm; ECIT: 0.98 ± 0.05 µm; *Z* = 0.59, *p* = 0.58, *n* = 17 releasing synapses from 5 animals) and major (Ctrl: 1.31 ± 0.10 µm; ECIT: 2.32 ± 0.16 µm; *Z* = 3.62, *p* < 0.001, *n* = 17 releasing synapses from 5 animals) full width at half maximums (**FWHMs**) of serotonin diffusion profiles at isolated releasing synapses before and after the bath application of ECIT. **F** Aspect ratio (Ctrl: 1.30 ± 0.06; ECIT: 2.02 ± 0.11; *Z* = 3.48, *p* < 0.001, *n* = 17 releasing synapses from 5 animals) and roundness (Ctrl: 0.79 ± 0.03; ECIT: 0.52 ± 0.03; *Z* = −3.53, *p* < 0.001, *n* = 17 releasing synapses from 5 animals) values of serotonin diffusion profiles at isolated releasing synapses before and after the bath application of ECIT. **G** Plots of pixel-wise maximal *Δ*F/F_0_ before (green) and after (red) the bath application of ECIT and their difference (cyan; ECIT-Ctrl). **H** Relative integration values of *Δ*F/F_0_ at distance of 0.5 µm (Ctrl: 0.37 ± 0.003; ECIT: 0.38 ± 0.003; *Z* = 1.34, *p* = 0.19, *n* = 17 releasing synapses from 5 animals), 0.5 − 3 µm (Ctrl: 0.49 ± 0.04; ECIT: 0.71 ± 0.05; *Z* = 2.91, *p* = 0.002, *n* = 17 releasing synapses from 5 animals), and 3 − 8 µm (Ctrl: 0.27 ± 0.04; ECIT: 0.38 ± 0.06; *Z* = 1.91, *p* = 0.06, *n* = 17 releasing synapses from 5 animals). Asterisks indicate *p* < 0.05 (Wilcoxon tests).

To understand how serotonin transporters influence serotonin after its release, we used GESIAP to analyze the spatial diffusion of released serotonin at synaptic, perisynaptic, and extrasynaptic areas, before and after bath application of escitalopram in the dorsal raphe nucleus (Fig. 6A). We typically identified individual releasing synapses with 10-pulse stimuli delivered at 64 Hz first, and then analyzed the spatial diffusion of serotonin released by 10-pulse stimuli delivered at 16 Hz. GESIAP created the serotonin spatial diffusion profiles with pixel-wise maximal *Δ*F/F_0_ plots, revealing that under the normal condition, serotonin diffusion at those well-isolated releasing synapses fit well with a single-exponential decay function that yielded a serotonin spread length constant of ~0.75 µm at raphe neurons (Fig. 6B, C). In the presence of escitalopram, pixel-wise maximal *Δ*F/F_0_ plots floated upwards at ~0.5 µm away from the releasing synapses (Fig. 6B, C). Arbitrarily fitting the plots with a single-exponential decay function gave an estimation of a larger serotonin spread length constant of ~0.85 µm at raphe neurons (Fig. 6C), indicating a significant effect of escitalopram on spatial diffusion of released serotonin.

The high-resolution serotonin spatial diffusion profiles created with GESIAP allowed us to zoom into those well-isolated releasing synapses, revealing that under the normal condition, serotonin concentration rapidly dropped as it diffused away from the release center, forming typically a circle cone-like profile with a full width at half maximum (**FWHM**) of ~0.5 µm, the size about synapses (Fig. 6D, E; Movie S2). Escitalopram enlarged the diffusion profiles and revealed 1–3 egress outlets that channeled serotonin for 3–5 µm towards the extrasynaptic space, which altered the profile to an ellipse cone-like sharp with a minor axis FWHM of ~0.5 µm and a major axis FWHM of ~1.0 µm, effectively doubling its aspect ratio and halving its roundness value (Fig. 6D–F). As a control, we found that repetitively evoking serotonin release did not change the serotonin diffusion profile and spread length constant at raphe neurons (Fig. S6). Quantitative analysis of the diffusion profiles revealed that escitalopram had little effect on serotonin accumulation and diffusion within the radius of ~0.5 µm from the release center (synaptic sites), enhanced serotonin accumulation and slowed its diffusion between the radii of ~0.5–3 µm from the release center (perisynaptic sites), and slightly increase serotonin buildup at ~3 µm or more from the release center (proximate extrasynaptic sites) (Fig. 6G, H). These results indicate the primary effect site of escitalopram to be ~0.5–3 µm from the release center, the perisynaptic area where serotonin transporters are highly expressed [38–40].

In the amygdalar preparation, we confirmed the impact of transporters on serotonin diffusion. Once more, the effect of escitalopram was evident in broadening the serotonin diffusion at GRAB_5HT_ expressing amygdalar neurons (Fig. S7A–C). This alteration transformed the diffusion profile from a circular cone-like shape to an elliptical cone-like form, revealing 1–3 egress channels through which serotonin traveled ~1–5 µm to reach the extracellular space (Fig. S7D–F). Specifically, escitalopram exhibited minimal effect on serotonin accumulation and diffusion within a radius of ~0.5 µm from the release center, the synaptic sites. However, it notably intensified serotonin accumulation and slowed its diffusion between radii of ~0.5–3 µm from the release center, the perisynaptic sites. Furthermore, it marginally increased serotonin buildup at distances of ~3 µm or more from the release center, the proximate extrasynaptic sites (Fig. S7G, H). Together, these results elucidate the impact of escitalopram on post-released serotonin diffusion in general.

We further verified the findings with the potent serotonin transporter inhibitor, paroxetine, in the mouse amygdalar preparation. Similar to escitalopram, paroxetine expanded serotonin diffusion at GRAB_5HT_ expressing amygdalar neurons, reshaping the diffusion pattern from a circular cone-like to an elliptical cone-like shape. This transformation revealed 1–3 pathways through which serotonin traveled ~1–5 µm before dispersing into the extracellular space (Fig. S8A–G). Once again, akin to escitalopram, paroxetine had minimal impact on serotonin accumulation and diffusion within a radius of ~0.5 µm from the release center, increased serotonin accumulation and slowed its diffusion between radii of ~0.5–3 µm from the release center, and marginally elevated serotonin buildup at distances of ~3 µm or more from the release center (Fig. S8H, I).

To eliminate the possibility of nonspecific involvement by other neurotransmitters, we conducted two sets of control experiments. First, bath application of inhibitors targeting AMPA receptors (**-Rs**), NMDA-Rs, GABA_A_-Rs, and GABA_B_-Rs, using NBQX, AP5, PTX, and CGP, respectively, showed no discernible influence on the serotonin diffusion profile (Fig. S9). Furthermore, we made in vivo AAV viral expression of Cre-dependent ChrimsonR-tdTomato in the dorsal raphe nucleus followed by Sindbis viral expression of GRAB_5HT_ in the amygdala of ePet-Cre mice, in which Cre recombinase is expressed under the serotonin transcription *FEV* promoter (Fig. S10A). Subsequently, we made optogenetic activation of serotonin fibers to induce selective release of serotonin at GRAB_5HT_ expressing amygdalar neurons in the ex vivo amygdalar slice preparation. Escitalopram induced a similar transformation in the serotonin diffusion pattern released via optogenetic stimulation, without affecting the amount of serotonin released or the number of releasing synapses (Fig. S10; Movie S3). Collectively, these findings support the general role of serotonin transporters in regulating serotonin diffusion subsequent to its release.

### Serotonergic transmission modes encode the properties of firing modes

**Fig. 7:**
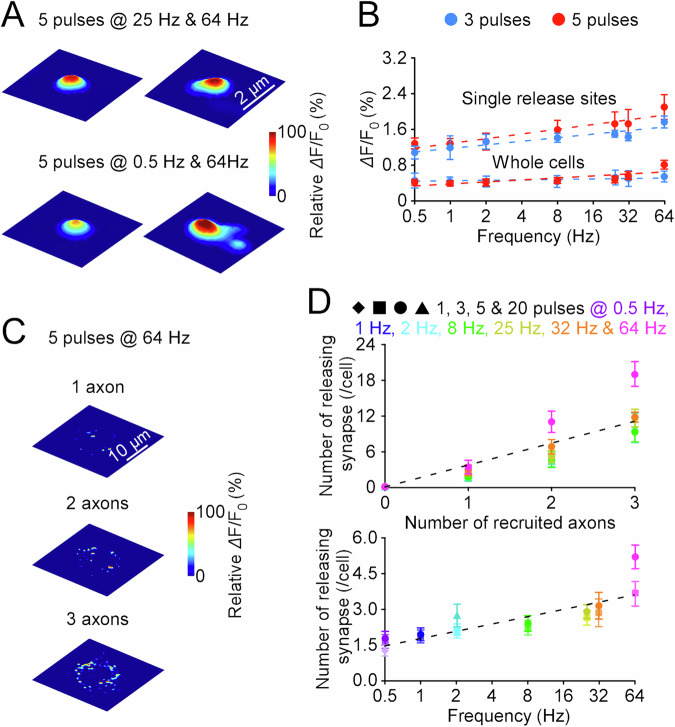
Serotonin release properties encode frequency, number, and synchrony of firing pulses. **A** Heatmaps of fluorescence *Δ*F/F_0_ responses electrically evoked by 5-pulse stimuli delivered at varied frequencies. Scale bars applied to all in **A**. **B** Plots of whole-cell and single releasing synapse maximal fluorescence *Δ*F/F_0_ responses against frequencies of stimuli containing 3 pulses (Whole cell: 0.5 Hz: 0.49 ± 0.15%; 1 Hz: 0.47 ± 0.09%; 2 Hz: 0.46 ± 0.12%; 8 Hz: 0.46 ± 0.13%; 25 Hz: 0.54 ± 0.17%; 32 Hz: 0.53 ± 0.18%; 64 Hz: 0.57 ± 0.13%, *n* = 5 neurons from 3 animals, fitted with log functions *f*_(*x*)_ = 0.47 + 0.013 • Log_2_ (x) (*r*^2^ = 0.60, *F* = 7.55, *p* = 0.04); Single releasing synapse: 0.5 Hz: 1.11 ± 0.15%; 1 Hz: 1.23 ± 0.26%; 2 Hz: 1.35 ± 0.16%; 8 Hz: 1.43 ± 0.10%; 25 Hz: 1.52 ± 0.08%; 32 Hz: 1.47 ± 0.09%; 64 Hz: 1.79 ± 0.13%, *n* = 5 releasing synapses from 3 animals, fitted with log functions *f*_(*x*)_ = 1.21 + 0.08 • Log_2_ (x) (*r*^2^ = 0.87, *F* = 33.64, *p* = 0.002)) and 5 pulses (Whole cell: 0.5 Hz: 0.40 ± 0.07%; 1 Hz: 0.38 ± 0.06%; 2 Hz: 0.41 ± 0.08%; 8 Hz: 0.44 ± 0.07%; 25 Hz: 0.46 ± 0.08%; 32 Hz: 0.55 ± 0.09%; 64 Hz: 0.80 ± 0.10%, *n* = 9 neurons from 5 animals, fitted with log functions *f*_(*x*)_ = 0.38 + 0.042 • Log_2_ (x) (*r*^2^ = 0.78, *F* = 7.57, *p* = 0.04); Single releasing synapse: 0.5 Hz: 1.29 ± 0.11%; 1 Hz: 1.29 ± 0.11%; 2 Hz: 1.33 ± 0.18%; 8 Hz: 1.58 ± 0.20%; 25 Hz: 1.73 ± 0.26%; 32 Hz: 1.71 ± 0.32%; 64 Hz: 2.09 ± 0.27%, *n* = 9 releasing synapses from 5 animals, fitted with log functions *f*_*(x)*_ = 1.30 + 0.10 • Log_2_ (x) (*r*^*2*^ = 0.89, *F* = 39.53, *p* = 0.002)). **C** Heatmaps of stepwise fluorescence *Δ*F/F_0_ responses electrically evoked by 5-pulse stimuli delivered at varied voltage intensities. Note the difference in number of releasing synapses in step 1–3 fluorescence *Δ*F/F_0_ responses. Scale bars applied to all in **C**. **D** Upper, plot correlating numbers of releasing synapses with presumably recruited axons. Note the correlation fitting with a linear function of *f*_(*x*)_ = 3.68 • *x* (*r*^*2*^ = 0.95, *F* = 53.23, *p* < 0.05). Lower, plot of numbers of releasing synapses against 1-, 3-, 5-, and 20-pulse stimuli delivered at 0.5 Hz (1 pulse: 1.30 ± 0.21, *n* = 5 from 3 animals; 3 pulses: 1.60 ± 0.40, *n* = 5 from 3 animals, 5 pulses: 1.77 ± 0.28, *n* = 9 from 5 animals), 1 Hz (3 pulses: 1.80 ± 0.2, *n* = 5 from 3 animals; 5 pulses: 1.92 ± 0.26, *n* = 9 from 5 animals), 2 Hz (3 pulses: 2.00 ± 0.45, *n* = 5 from 3 animals; 5 pulses: 2.15 ± 0.22, *n* = 9 from 5 animals; 20 pulses: 2.72 ± 0.48, *n* = 9 from 5 animals), 8 Hz (3 pulses: 2.20 ± 0.37, *n* = 5 from 3 animals; 5 pulses: 2.39 ± 0.31, *n* = 9 from 5 animals), 25 Hz (3 pulses: 2.60 ± 0.40, *n* = 5 from 3 animals; 5 pulses: 2.88 ± 0.30, *n* = 9 from 5 animals), 32 Hz (3 pulses: 2.80 ± 0.37, *n* = 5 from 3 animals; 5 pulses: 3.10 ± 0.56, *n* = 10 from 5 animals), 64 Hz (3 pulses: 3.60 ± 0.40, *n* = 5 from 3 animals; 5 pulses: 5.16 ± 0.50, *n* = 10 from 5 animals), fitted with a linear function of *f*_(*x*)_ = 0.31 • *x* + 1.75 (*r*^*2*^ = 0.79, *F* = 18.62, *p* < 0.01).

The preceding data underscore the substantial impact of serotonin release and its interaction with transporters in serotonin signaling. This prompted our investigation to delve systematically into how diverse modes of serotonergic transmission encode distinct characteristics of various firing modes within raphe serotonergic neurons (Fig. 7). As we increased the frequency of electric stimuli, we observed a corresponding escalation in evoked fluorescence *Δ*F/F_0_ responses, revealing a subtle positive linear relationship with the logarithmically scaled stimulation frequencies (Fig. 7A, B; Movie S4). Additionally, the evoked fluorescence *Δ*F/F_0_ responses of single releasing synapses showed a modest elevation with higher stimulation pulse numbers (Fig. 7A, B). These results align with the observed high release probability of serotonergic transmission. Moreover, the numbers of serotonin releasing synapses showed a robust correlation with the numbers of synchronously activated axons (Fig. 7C, D; Movie S5), uncovering an additional layer to the coding mechanism. Finally, the numbers of serotonin releasing synapses exhibited a weak positive correlation with stimulation frequencies, but not with pulse numbers (Fig. 7D), representing a third facet of the coding scheme. Taken together with the analysis of synaptic and volume transmission, these results shed light on how serotonin utilizes neurotransmitter quantity and releasing synapse count to encode the frequency, number, and synchronicity of firing pulses, while employing additional volume transmission to signal heightened firing activities.

## Discussion

This study introduces a sensor-based imaging and FSCV method, enabling simultaneous recordings of synaptic, perisynaptic, proximate, and distal extrasynaptic serotonergic transmission for the first time. This method offers insights into the diverse modes and regulation of serotonin communication. Our key discovery highlights how various firing patterns of serotonergic neurons create distinct transmission modes. These transmission modes encode details about firing frequency, pulse number, and synchrony in neurotransmitter quantity, releasing synapse count, and the involvement of synaptic and/or volume transmission. These findings emphasize the intricate collaboration of synaptic structure, properties, and transporters in orchestrating the spectrum of serotonin transmission modes.

### Serotonergic transmission modes

This study delineates a spectrum of serotonergic transmission modes. Serotonergic neurons participate in a wide array of behaviors, spanning cognition, mood, social interaction, sexual behavior, feeding behavior, motor behavior, reward, motivation, sleep-wake cycle, and thermoregulation [2, 3, 41]. Corresponding to this wide range of functions, serotonergic neurons exhibit a diverse spectrum of firing patterns [4–9]. Yet, despite this connection, unraveling how these varied firing patterns impact serotonin release and signaling, subsequently influencing associated behaviors, has remained an enduring mystery [3, 10]. Here, we introduce an innovative method allowing for nanoscopic visualization of individual releasing synapses during transmitter release. Our analysis demonstrates that serotonergic neurons intricately encode the frequency, number, and synchronization of their firing pulses, converting these parameters into the quantity of serotonin released and the count of releasing synapses. Particularly noteworthy is the revelation that high-frequency and synchronized firing patterns overwhelm serotonin transporters, causing serotonin to disperse into the extracellular space and instigate volume transmission. Consequently, by modulating transmitter quantity, releasing synapse count, and employing synaptic and/or volume transmission, serotonergic neurons effectively convert diverse firing modes into distinct intercellular signals. The complexity of these signals has the potential to transmit heterogeneous firing patterns to varied spatiotemporally defined G-protein coupled receptor signaling in postsynaptic targets, initiating a broad range of functions [42, 43].

### Conversion of synaptic to volume transmission via channeled diffusion

Our analysis offers new insights into another enduring debate on serotonergic transmission. The prevailing volume transmission theory posits that neuromodulatory transmitters, like serotonin, diffuse extensively across long distances, influencing various nearby cells [12, 13]. However, this theory relies on the assumption that endogenously released neuromodulators behave akin to exogenously applied ones, freely diffusing in the extrasynaptic space—an assumption lacking direct experimental support [44, 45]. Recent studies employing improved voltammetry challenge this view, suggesting a serotonin diffusion range of ~20 µm or slightly more, resembling paracrine-like transmission [46, 47]. Nonetheless, due to voltammetry’s limitations in measuring solely extracellular serotonin, these studies do not have spatial resolution to directly examine serotonergic transmission at synapses [11]. Conversely, an electrophysiology study suggests that serotonergic transmission primarily occurs via synaptic mechanism with minimal crosstalk between synapses [14]. However, this study, reliant on indirect analysis, struggles to explain observed evidence supporting serotonin spillover in some experiments. Here, our imaging analysis presents the first nanoscopic visualization of highly confined diffusion of serotonin at individual releasing synapses, supporting the presence of tightly restricted synaptic serotonergic transmission as a typical mode of signaling, with volume transmission occurring under specialized conditions [34].

In this study, our comprehensive high-resolution imaging analysis reveals serotonin’s diffusion dynamics. At synapses, serotonin diffuses freely in a distinctive cone-shaped pattern within a radius of ~0.5 µm from the release site. However, at perisynaptic and proximate extrasynaptic regions, serotonin is rapidly cleared by transporters, causing a steep concentration drop, often leaving only a minimal concentration undetectable by FSCV_5HT_ at distal extrasynaptic sites. During heightened release or under transporter inhibition, a slightly larger quantity manages to escape synapses through one, sometimes two, or occasionally three outlets. While this increased release remains below GRAB_5HT_ detection threshold at proximate extrasynaptic sites, it becomes detectable at distal extrasynaptic sites by FSCV_5HT_. This hints a volume transmission, mediated for example by the high affinity serotonin receptors expressed in certain brain areas [48]. The outlets exposed to transporter inhibition suggest the existence of channeled synaptic enclosures.  This is consistent with ultrastructural observations of numerous clusters of curved gap junctions, which form annular or tapered annular profiles with diameters ranging from ~2–7 µm at serotonergic perisynaptic sites [39, 49]. These channeled synaptic enclosures likely play a significant role in dispersing serotonin into extracellular sites, particularly by governing the diffusion direction of serotonin via 1–3 exiting channels. Nevertheless, owing to the spatial resolution limitations of FSCV_5HT_, it remains to determine whether serotonin diffuses freely or through specific channels that are physically restricted by cellular structures and/or regulated by transporters (see [40]) at the distal extracellular space.

Based on the serotonin concentration-fluorescence response curve obtained from the perfusion experiment, we estimated that ~100 nM of serotonin is released per quantal event. At these concentrations, fewer than one serotonin molecule would be present in the cleft volume of serotonergic synapses (~0.6–0.8 µm in diameter; [50–52]), which seems implausible. Previous studies suggest that serotonin 2C receptors may exhibit ~25–50 times lower affinity at synapses [37, 53, 54]. Since synaptic GRAB_5HT_ sensors share similar pharmacological properties with serotonin 2C receptors [55], their affinity at synapses may also be significantly reduced. Consequently, synaptic serotonin concentrations might be underestimated by a similar factor, implying that ~2.5–5 mM of serotonin could be released per quantal event. Directly measuring the synaptically evoked serotonin concentration-fluorescence response, as previously demonstrated by Marcott and colleagues [56], is essential for accurately calibrating the vesicular quantal size and validating this study’s main conclusions.

### Biological and clinical implication

Intercellular communication, which is mediated by *fast*-acting transmitters glutamate and gamma-aminobutyric acid (**GABA**), and by hundreds (>500) of *slow*-acting neuromodulatory transmitters (e.g., monoamines, neuropeptides, and other molecules), orchestrates diverse behavioral and physiological processes [57, 58]. A wealth of knowledge has been acquired thanks to the remarkable sensitivity and temporal resolution of electrophysiological recordings, as well as through Herculean experiments examining fast neurotransmitters [59–61]. It is worth noting that in the early 1990s, the advent of high-resolution patch-clamp recordings enabled the quantitative analysis of glutamatergic and GABAergic synaptic transmissions and their properties [62–64]. The technique sparked a significant wave of discoveries concerning synaptic transmission and plasticity. These breakthroughs include silent synapses, synaptic receptor trafficking, synaptic molecular signaling, synaptic nanocolumnar organization, and many other vital insights crucial for comprehending the brain, body, associated disorders, and ultimately, for identifying pathogenic mechanisms and therapeutic strategies [58, 65–67]. Unfortunately, patch-clamp recordings fall short in effectively capturing neuromodulatory transmission properties. Other methods, like microdialysis and voltammetry, despite many recent improvements, still lack vital spatial and/or temporal resolution [11]. Therefore, despite extensive research linking slow-acting neuromodulatory transmitters to diverse physiological actions and diseases, a significant gap persists, hindering comprehensive understanding of neuromodulatory regulation and function. The emergence of genetically encoded fluorescence sensors, many of which emit abundant photons, presents a promising avenue. Our theoretical groundwork suggests that employing image analysis algorithms could potentially convert the surplus collected photons into enhanced spatial and temporal resolution, offering a means to decode neuromodulatory transmission [68, 69].

Recently developed image analysis program, GESIAP, has demonstrated its utility in delineating fundamental synaptic properties at individual serotonin releasing synapses [31, 35]. This includes aspects like transmitter spatial diffusion, quantal size, quantal content, release probability, pool size, and refilling rate. Here, we show the ability of GESIAP to enable nanoscopic visualization of serotonin diffusion across various behavior-related activity patterns. This reveals how channeled synaptic enclosures, synaptic properties, and transporters collaboratively shape serotonergic transmission modes. This mechanism explains why SSRIs may elevate extracellular serotonin levels, as observed in voltammetry [18–21], without affecting serotonin release at synapses. More importantly, the findings predict that the alterations in serotonin transporters, whether they occur due to physiological, pathological, or clinical conditions (e.g., see [1, 70]; see also [41]), as well as changes in synaptic structure and properties (our unpublished data), hold the potential to influence behaviors and contribute to the development and prognosis of various diseases. We observe that fundamental synaptic properties of serotonergic transmission are largely conserved across different brain areas (e.g., the thalamic geniculate, dentate gyrus, dorsal raphe, and amygdala), animal species (e.g., mouse, rat, and human), and preparations (e.g., cultured neuron systems, ex vivo, and intact brains) (this study; [31, 34]; our unpublished data). Therefore, the method reported herein, which enables effective quantification of synaptic changes and transporter functions, promises to provide insights into synaptic mechanisms involved in a range of serotonin-linked diseases, e.g., major depressive disorders, digestive disorder, obsessive-compulsive disorder, sexual dysfunction, sleep disorder, and social anxiety disorder [1–3].

## Methods

### Animal preparations

Wild type C57BL/6J mice (Jackson Laboratory, Bar Harbor, ME; RRID:IMSR_JAX:000664), ePet-Cre mice (Jackson Laboratory; Stock #012712; RRID:IMSR_JAX:012712) bred congenically on the C57BL/6J background [71], and Sprague Dawley rats were utilized in this study. Genotyping was conducted through standard PCR using genomic DNA extracted from tail samples, employing transgene primers: 5′-GCG GTC TGG CAG TAA AAA CTA TC-3′ (Forward) and 5′-GTG AAA CAG CAT TGC TGT CAC TT-3′ (Reverse), and internal positive control primers 5′-CTA GGC CAC AGA ATT GAA AGA TCT-3′ (Forward) and 5′-GTA GGT GGA AAT TCT AGC CAT CAT C-3′ (Reverse). Heterozygous ePet-Cre mice exhibiting specific DNA bands at both transgene and internal positive control positions were included in the experiments.

Both male and female animals were included and neither genetically encoded sensor-based imaging nor fast-scan cyclic voltammetry (**FSCV**) detected any difference in serotonergic responses measured in tissues prepared from males versus females. Animals were maintained in the animal facility at the University of Virginia and family- or pair-housed in the temperature-controlled animal room with 12-h/12-h light/dark cycle. Food and water were available *ad libitum*. All procedures for animal and human cell line experiments were performed following protocols (No. 3168) approved by the Animal Care & Use Committee of the University of Virginia and in accordance with US National Institutes of Health guidelines.

### Cultured and acute slice preparations

Organotypic hippocampal cultured slices were prepared from postnatal 5–7 day old male/female Sprague Dawley rats, following our previous studies [72]. Briefly, the hippocampi were dissected out in ice-cold HEPES-buffered Hanks’ solution (pH 7.35) under sterile conditions, sectioned into 400 µm slices on a tissue chopper, and explanted onto a Millicell-CM membrane (0.4-µm pore size; Millipore, Billerica, MA). The membranes were then placed in 750 µl of MEM culture medium, contained (in mM): HEPES 30, heat-inactivated horse serum 20%, glutamine 1.4, D-glucose 16.25, NaHCO_3_ 5, CaCl_2_ 1, MgSO_4_ 2, insulin 1 mg/ml, ascorbic acid 0.012% at pH 7.28 and osmolarity 320. Cultured slices were maintained at 35 °C, in a humidified incubator ambient air enriched with 5% CO_2_.

Acute dorsal raphe and amygdalar slices were typically prepared from P42 − 60 male/female C57BL/6 J mice ~18 h after in vivo viral expression of genetically encoded serotonin sensor as our previous reports [25, 31]. Animals were deeply anesthetized by xylazine-ketamine and decapitated. The brain containing the dorsal raphe nucleus or the amygdala or the central nucleus of the amygdala was quickly removed and placed into cold (0–4 °C) oxygenated physiological solution containing (in mM): NaCl 125, KCl 2.5, NaH_2_PO_4_ 1.25, NaHCO_3_ 25, MgCl_2_ 1, dextrose 25, and CaCl_2 _2, pH 7.4. Coronal slices 350 μm thick were cut from the brain blocks. These slices were kept at 37.0 ± 0.5 °C in oxygenated physiological solution for ~0.5–1 h before sensor imaging and FSCV recordings.

During imaging and FSCV recordings, the brain slices were submerged in a chamber and stabilized with a fine nylon net attached to a platinum ring. The recording chamber was perfused with oxygenated physiological solution. The half-time for the bath solution exchange was ~6 s, and the temperature of the bath solution was maintained at 34.0 ± 0.5 °C. All antagonists were bath-applied.

### GRAB_5HT_ preparation and expression

Genetically encoded serotonin fluorescent sensors GRAB_5HT1.0_ and iSeroSnFR were sub-cloned into Sindbis viral vector pSinREP5 with Xba1 and Sph1 restriction digestion, and viral particles were produced and expressed following our previous studies [25, 27]. For in vitro sensor expression, a glass pipette was used to deliver ~10 nl of Sindbis solution by pressure injection to infect CA1 pyramidal neurons in cultured hippocampal slices. Experiments were typically performed within 18 ± 4 h of Sindbis viral infection.

For in vivo sensor expression, animals were initially anesthetized by an intraperitoneal injection of ketamine and xylazine (10 and 2 mg/kg, respectively), and then placed in a stereotaxic frame. A glass pipette was used to penetrate the dorsal raphe nucleus (**DR**) and the central nucleus of the amygdala (**CeA**) according to stereotaxic coordinates (DR: AP −4.35 mm, ML: 0.00 mm, DV: −3.20 mm; CeA: AP −1.50 mm, ML: ±2.50 mm, DV: −4.70 mm) to deliver ~50 nl of Sindbis solution by pressure injection to infect raphe neurons. For in vivo opsin expression, 80 nl of AAV-Syn-FLEX-ChrimsonR-tdTomato viral solution (Addgene, Watertown, MA; RRID:Addgene_62723) was injected into DR of ePet-Cre mice. The in vivo viral injection micropipette was typically left in the brain for ~10 min after the injection to ensure proper viral diffusion. Experiments were typically conducted within 18 ± 4 h after Sindbis viral infection and 5‒6 weeks after AAV viral infection to achieve optimal expression [73, 74].

### Electric and optogenetic activation of serotonin release

Serotonin release was triggered using either electrical or optogenetic approaches. To evoke serotonergic responses through electrical stimulation, a bipolar cluster stimulating electrode (CE2C65; FHC, Bowdoin, ME) was typically employed and positioned near either the dorsal raphe nucleus or the amygdala. The stimulation current varied between ~100–350 µA, with each pulse lasting 1 ms. Except in the case of minimal stimulation experiments designed to evaluate the incremental recruitment of activated axons, suprathreshold stimulation was typically administered to ensure the activation of serotonergic fibers, as confirmed by consistent whole-cell fluorescence responses. The evoked fluorescence responses were sensitive to bath application of TTX, 0 mM Ca^2+^/10 mM Mg^2+^, calcium channel blockers, and pharmacological inhibitors of sensors (Fig. S3; see also [25, 27, 28, 31, 33, 34]), confirming synaptic responses.

To optogenetically induce serotonin release, a 200 μm diameter optical fiber (Thorlabs, Newton, NJ) was positioned near CeA along the serotonergic fiber pathway to activate ChrimsonR (Cre-dependent) using a 635-nm laser (Opto Engine LLC, Midvale, UT). The stimulation protocol consisted of five pulses, each lasting 10 ms, delivered at 64 Hz, with the light intensity set to 10 mW/mm² at the fiber tip. Additionally, 200 µM 4-AP was included into the bath solution to facilitate optogenetic activation. Moreover, to prevent unintended opsin activation during the simultaneous GRAB_5HT_ sensor-based imaging, a low sensor excitation light intensity of 0.07 mW/mm² was typically used in these experiments. Furthermore, due to the high power required for optogenetic activation, only low frequency and low intensity stimulation was applied in this study. To avoid interference of the optogenetic excitation light with GRAB_5HT_ fluorescence responses, 2‒3 frames of images obtained during optogenetic stimulation were excluded from the analysis.

### Sensor-based functional imaging

Due to the slow nature of neuromodulatory transmission, long-term imaging (from seconds to minutes) became necessary to capture the action of transmitters and resolve their properties. To minimize drift and fluctuation vital for high-resolution visualization of transmitter release-induced fluorescence responses [68], a stable recording/stimulation and imaging setup was used to carry out all imaging and FSCV recording experiments [33, 75]. Wide-field epifluorescence imaging was performed using a Hamamatsu ORCA FLASH4.0 camera (Hamamatsu Photonics, Shizuka, Japan), and fluorescent sensor expressing cells in acutely prepared tissue slices were excited by a 460-nm ultrahigh-power low-noise LED (Prizmatix, Givat-Shmuel, Israel) [27, 75]. The frame rate of the FLASH4.0 camera was set to 10–50 Hz. Fluorescence signals were collected with an Olympus 40× water-immersion objective with a numerical aperture of 0.8. Despite variable fluorescence F_0_ across the entire cell membrane surface of neurons due to heterogeneous GRAB_5HT_ sensor expression, the *Δ*F/F_0_ responses showed no or weak correlation with the basal fluorescence F_0_, suggesting independence from sensor expression levels and reliability in measuring transmitter concentration (Fig. S11; cf. [31, 34]).

### Imaging analysis with GESIAP

Recent advancements in computational algorithms technology have spurred a swift expansion of applications for superresolution wide-field deconvolution microscopy [76–79], which can achieve ~150–200 nm spatial resolution, sufficient to resolving synapses, including serotonergic synapses sizing ~0.6–0.8 µm in diameters [50–52]. We have previously validated its applicability in sensor-based functional imaging, both theoretically [68] and experimentally [34]. In this study, fluorescence responses were analyzed using enhanced algorithms derived from our recently created genetically encoded sensor-based image analysis program (**GESIAP**) [31]. The improved program (i.e., GESIAP3.0 codes [35], generously shared by Ke Si, Jiazhu Zhu, and Roger R. Zhu, Zhejiang University) proved to be was effective in extracting small fluorescence responses from individual releasing synapses [68], essential for delineating synaptic properties. The algorithms were created using MATLAB 2023a with MATLAB’s Image Processing Toolbox (Mathworks, Natick, MA). The fluorescence response traces typically represented the whole-cell average responses of individual neuronal somata of interest, unless stated otherwise (e.g., single releasing synapses on the soma and dendrite). To visualize individual transmitter releasing synapses and estimate postsynaptic transmitter spatial diffusion extent, the maximal electrically evoked maximal *Δ*F/F_0_ responses at individual pixels over time were plotted to create 3D spatial profiles for individual releasing synapses. Pixels with the maximal *Δ*F/F_0_ responses in individual releasing synapses were assumed to be the centers of release. Fluorescence *Δ*F/F_0_ intensity profiles at well-isolated releasing synapses were fit with a single-exponential decay function, and their decay constants were extracted as the spatial spread length constants. We found that higher-order decay functions, such as two-exponential decay functions, provided only limited improvement in fitting both the control and SSRI conditions, and therefore opted for one-exponential fits for simplicity. To estimate the quantal properties of individual transmitter releases, 20-pulse trains at low frequency (0.1 Hz) were used to evoke transmitter release. The evoked fluorescence responses at isolated individual releasing synapses were fit with a double-exponential synaptic function incorporating the response properties of GRAB_5HT_, using MATLAB algorithms in GESIAP generously shared by Roger R. Zhu [34, 35]. This provided estimates of key parameters of the evoked events such as rise time, decay time constant, and amplitude. The quantal properties, such as quantal content, quantal size, and release probability, were determined using the classic quantal analysis approach [62–64].

### Fast-scan cyclic voltammetry

Carbon-fiber microelectrodes (**CFME**) were fabricated from 7 µm T-650 carbon fibers (Cytec Engineering Materials Inc, Tempe, AZ), which were aspirated into a glass capillary (1.2 mm OD and 0.68 mm ID, A-M System, Sequim, WA) and pulled into electrodes with a PE-22 puller (Narishige International USA Inc, Amityville, NY). The carbon fiber was trimmed to 50 to 70 μm in length from the pulled glass tip, and then sealed with Epon epoxy cured at 100 °C for 2 h followed by 150 °C overnight. CFMEs were cleaned in isopropyl alcohol for 30 min, and then electrochemically deposited by submerging CFME tip in Nafion® solution (5 wt% 1100 EW Nafion® in methanol, Ion Power, New Castle, DE) with a constant potential of 1.0 V vs Ag/AgCl applied to them for 30 s [80]. The Nafion-coated electrodes were air-dried for 10 s, and then at 70 °C for 10 min. For electrochemical detection of 5HT, a Jackson waveform was applied to the electrode by scanning the potential cycling from 0.2 → 1.0 → −0.1 → 0.2 V at 1000 V/s using a Dagan ChemClamp potentiostat (Pine Research Instrumentation, Durham, NC). Due to the potential eletrode fouling [81], the observed enhanced FSCV_5HT_ responses might be understimated in the presenece of SSRIs. For data collection and analysis, TarHeel CV (generously provided by R. Mark Wightman, University of North Carolina) was used. For the electrode calibrations, phosphate buffer solution was used which consisting of (in mM): NaCl 131.25, KCl 3.0, NaH_2_PO_4_ 10.0, MgCl_2_ 1.2, Na_2_SO_4 _2.0, and CaCl_2 _1.2, at pH 7.4. A serotonin stock solution was prepared in 0.1 M HClO_4_ and diluted to 500 nM with phosphate buffer solution for calibrations prior to the experiment.

### Experimental operation and data collection

The experiments were operated under control of a single custom-written program based on IGOR (WaveMetrics, Lake Oswego, OR) for electrophysiology, optogenetics, and functional imaging [75, 82]. This program synchronized image capture, voltammetry, electric or optogenetic stimulations, and comprehensive data collection. The voltammetric FSCV_5HT_ response recording started typically 10 s after GRAB_5HT_ imaging to avoid the potential blue light-induced photoelectric effect on the measurement of evoked voltammetric responses [83].

### Statistical analysis

Statistical results were reported as mean±s.e.m. Animals or cells were randomly assigned into control or experimental groups and investigators were blinded to experimental treatments. Based on the effect size *d* calculated from previous and preliminary data, the sample size is estimated to be ≥~8–25/group to attain the desired power of ≥80% with statistical significance set as α < 0.05. Statistical significance of the means is determined using Wilcoxon and Mann-Whitney Rank Sum non-parametric tests for paired and unpaired samples, respectively. Statistical significance of the relationship of two data groups is determined using regression *t* test provided the normality and constant variance tests passed. The data that support the findings of this study are available from the corresponding authors upon request.

## Supplementary information

Supplemental materials

## Data Availability

The authors confirm that the data supporting the findings of this study are available within the article and its supplementary materials. All relevant data have been presented in this article. There was no data excluded from the analysis.
