# Supplementary material for: Coding principles and mechanisms of serotonergic transmission modes
Source: Mol Psychiatry. 2025 Feb 22;30(8):3430–42. doi: 10.1038/s41380-025-02930-4 (PMC12240840; doi:10.1038/s41380-025-02930-4)

Supporting Information for

Coding principles and mechanisms of serotonergic transmission modes

Running title: Nanoscopic visualization of serotonergic transmission at synapses

by

Yajun Zhang, Peng Zhang, Mimi Shin, Yuanyu Chang, Stephen B. G. Abbott, B. Jill Venton and J. Julius Zhu

Figure S1

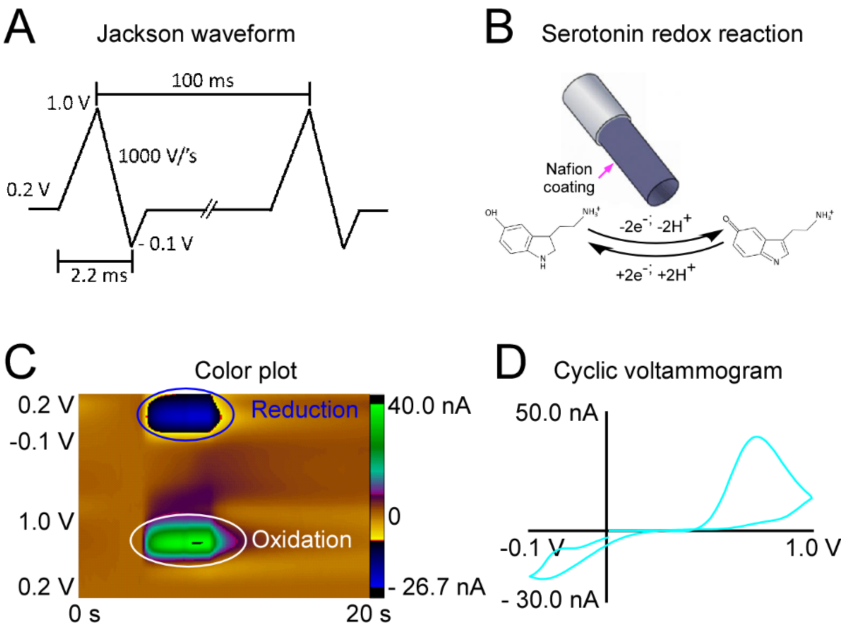

Figure S1. FSCV$_{5HT}$ enables measurement of serotonin-specific signals.

(**A**) Jackson waveform, a serotonin specific waveform, was used to selectively measure serotonin.

(**B**) Serotonin undergoes redox reaction as the Jackson waveform was applied to a Nafion-coated carbon-fiber microelectrode.

(**C**) Pseudo-color plot shows oxidation (green) and reduction (blue) for serotonin.

(**D**) Cyclic voltammogram extracted from the vertical line of pseudo-color plot shows oxidation (0.7 V) and reduction (-0.1 V) peaks for serotonin.

Figure S2

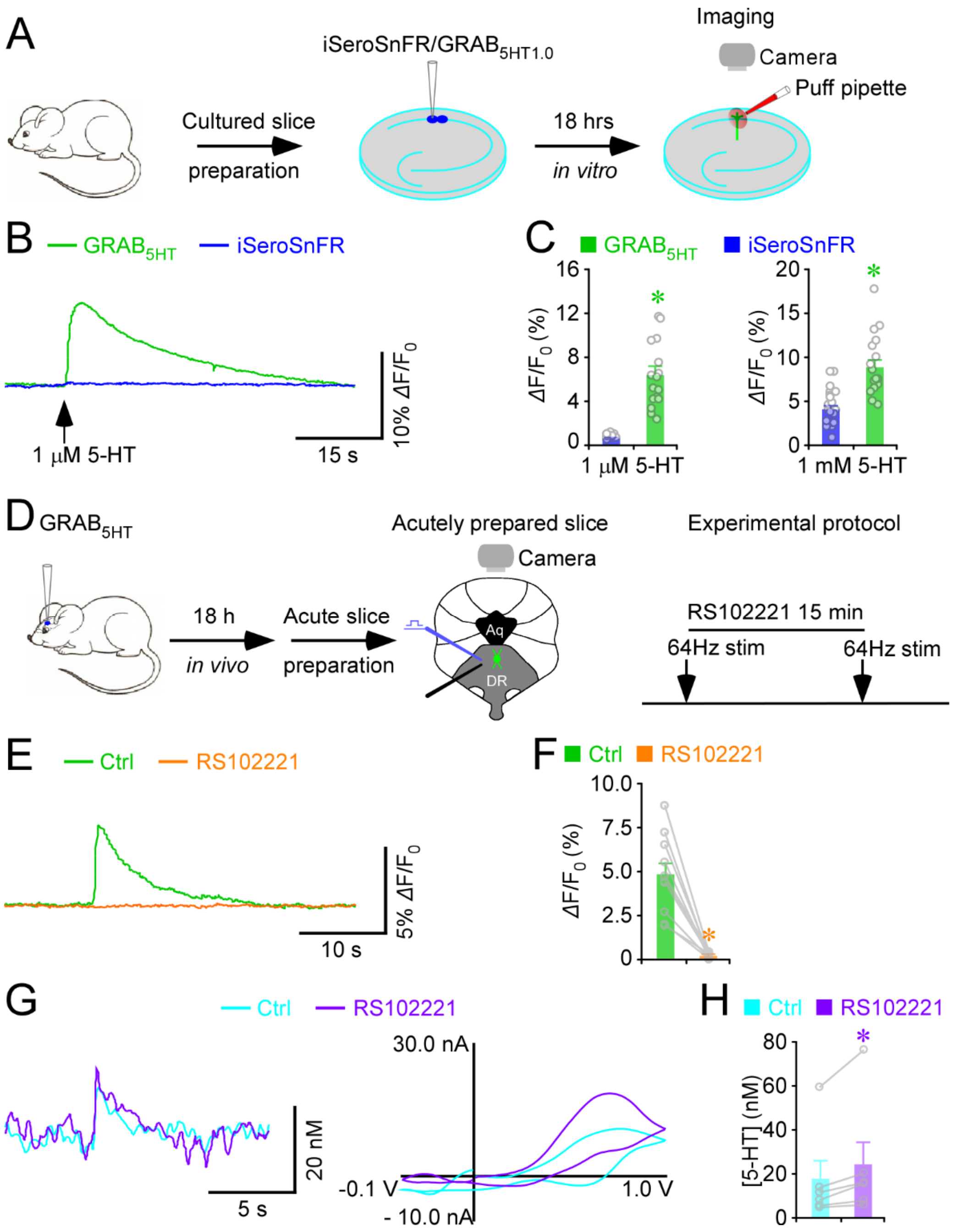

Figure S2. GRAB$_{5HT}$ enables measurement of serotonin-specific signals.

(**A**) Schematic of imaging experiment design in a cultured rat hippocampal slice preparation.

(**B**) Fluorescence *Δ*F/F$_0$ responses of GRAB$_{5HT}$ or iSeroSnFR expressing CA1 pyramidal neurons in response to 10-ms puffs of 1 μM 5-HT.

(**C**) Peak *Δ*F/F$_0$ responses of GRAB$_{5HT}$ and iSeroSnFR expressing CA1 neurons to puff of 1 μM (GRAB$_{5HT1.0}$: 5.93 ± 0.71%; iSeroSnFR: 0.60 ± 0.04%; U = 400, n = 20 neurons from 8 animals, p < 0.001) and 1 mM

($GRAB_{5HT1.0}$: 8.81 ± 0.86%; iSeroSnFR: 4.00 ± 0.39%; U = 36, n = 17 neurons from 8 animals, p < 0.001) 5-HT. Asterisks indicate p < 0.05 (Mann-Whitney Rank Sum tests).

(**D**) Schematic of multiplexed imaging and voltammetric experiment design in a mouse dorsal raphe nucleus (**DR**) slice preparation.

(**E**) Fluorescence $\Delta F/F_0$ responses of $GRAB_{5HT}$ expressing raphe neurons evoked a train of 20 electric pluses delivered at 64 Hz in the normal control bath solution and bath solution containing 10 μM RS102221.

(**F**) Peak $\Delta F/F_0$ responses in the control bath solution and bath solution containing 10 μM RS102221 (Ctrl: 4.80 ± 0.64%; RS102221: 0.26 ± 0.05%; Z = 2.93, p < 0.001, n = 11 neurons from 3 animals).

(**G**) $FSCV_{5HT}$ current traces and cyclic voltammograms in the normal control bath solution and bath solution containing 10 μM RS102221.

(**H**) Peak serotonin concentrations in the control bath solution and bath solution containing 10 μM RS102221 (Ctrl: 17.67 ± 8.43 nM; RS102221: 24.03 ± 10.60 nM; Z = 2.2, p = 0.04, n = 6 neurons from 3 animals). Asterisks indicate p < 0.05 (Wilcoxon tests).

Figure S3

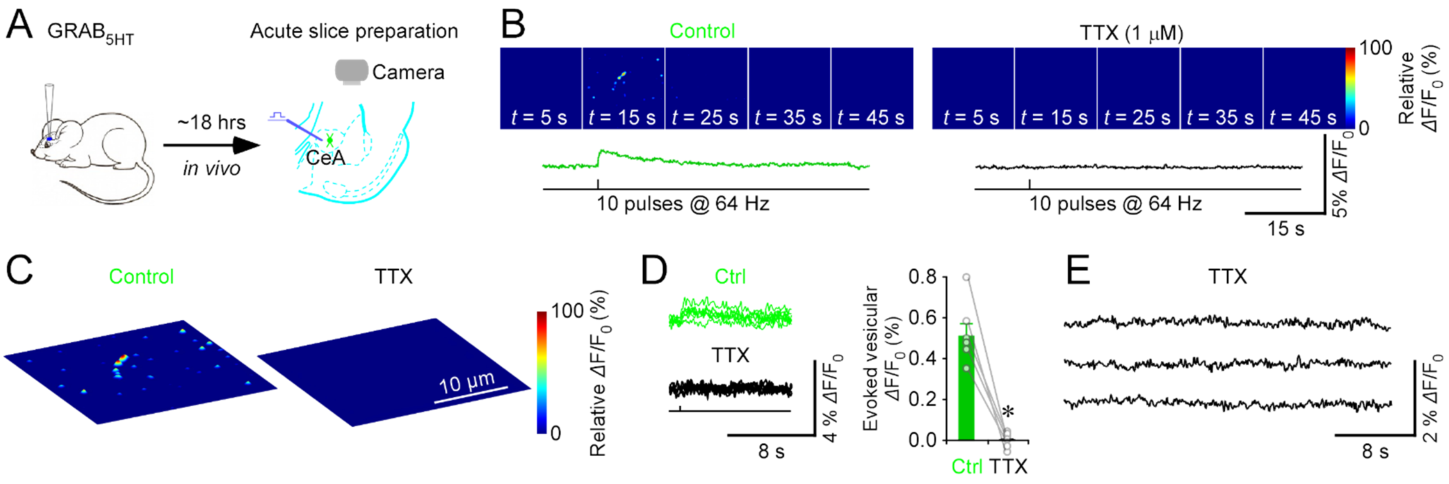

Figure S3. TTX diminishes the evoked $\Delta F/F_0$ responses at $GRAB_{5HT}$ expressing neurons.

(**A**) Schematic of experiment design in a mouse amygdalar slice preparation. CeA: the central nucleus of the amygdala.

(**B-C**) Heatmaps and 3D spatiotemporal profiling of electrically evoked fluorescence $\Delta F/F_0$ responses before (left) and after (right) the bath application of 1 μm Tetrodotoxin (**TTX**) in the amygdala. Scale bars applied to all in **B-C**.

(**D**) Left, $\Delta F/F_0$ responses evoked by single pulse stimuli at the isolated releasing synapses before (green) and after (black) the bath application of TTX. Right, evoked vesicular responses (Ctrl: 0.53 ± 0.06%; TTX: 0.0075 ± 0.015%; Z = -2.20, p = 0.03, n = 6 neurons from 5 animals). Asterisks indicate p < 0.05 (Wilcoxon tests).

(**E**) Fluorescence recordings of the $GRAB_{5HT}$ expressing neuron show no spontaneous release in the presence of TTX.

Figure S4

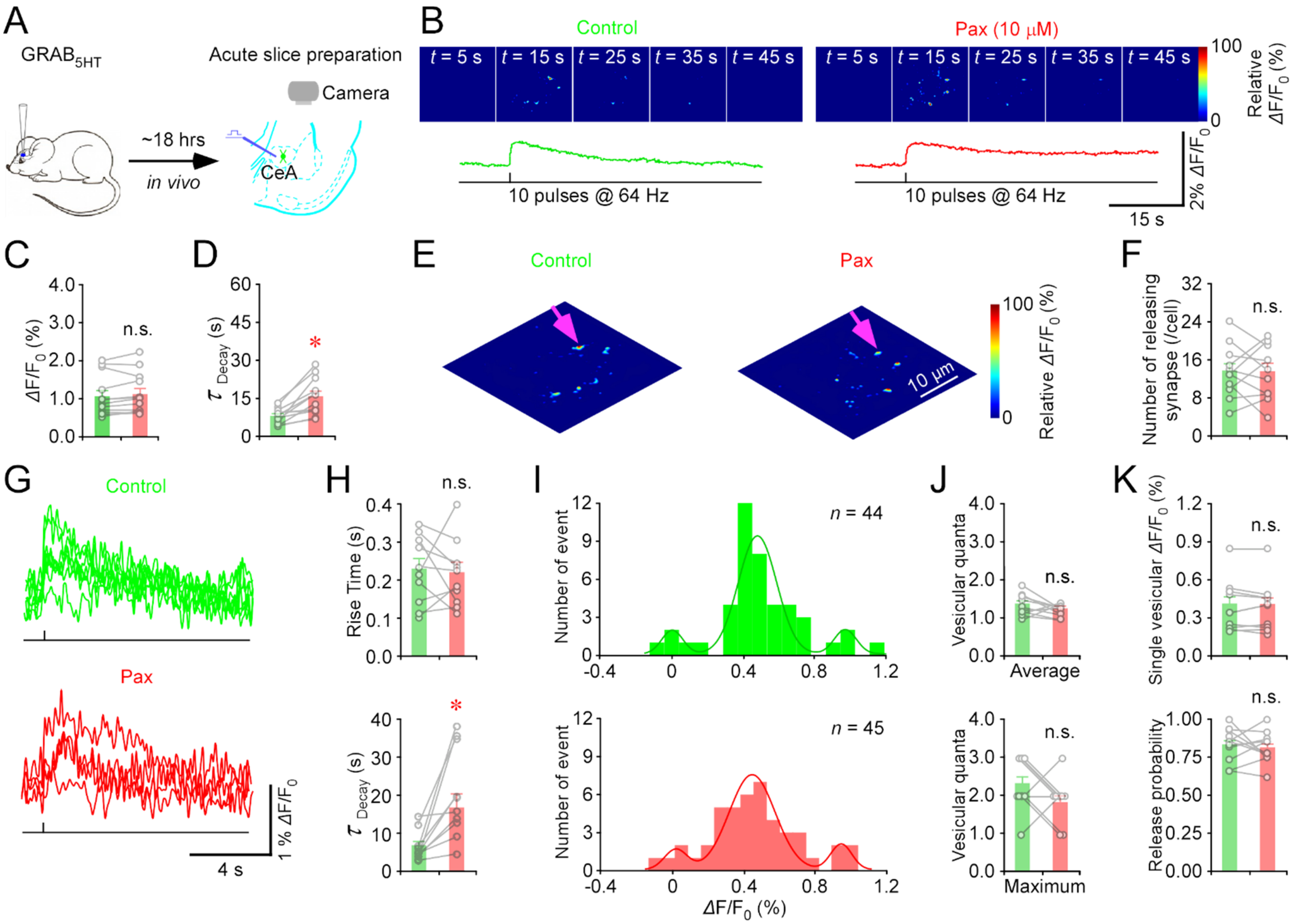

Figure S4. Transporter inhibitor paroxetine prolongs amygdalar serotonin signals without affecting release.

(**A**) Schematic of experiment design in a mouse amygdalar slice preparation. CeA: the central nucleus of the amygdala.

(**B**) Heatmaps of electrically evoked fluorescence $\Delta F/F_0$ responses before (left) and after (right) the bath application of 10 μm paroxetine (**Pax**) in the amygdala.

(**C-D**) Peak amplitudes (Ctrl: 1.08 ± 0.15%; Pax: 1.13 ± 0.15%, $Z = 1.64$, $p = 0.11$, $n = 12$ neurons from 6 animals) and decay time constants (Ctrl: 8.07 ± 0.89 s; Pax: 15.87 ± 2.05 s, $Z = 3.06$, $p = 0.003$, $n = 12$ neurons from 6 animals) $GRAB_{5HT}$ fluorescence $\Delta F/F_0$ responses before (green) and after (red) the bath application of Pax.

(**E**) 3D spatiotemporal profiling of electrically evoked fluorescence $\Delta F/F_0$ responses before (left) and after (right) the bath application of Pax. Scale bars applied to all in **B** and **E**.

(**F**) Releasing synapse counts before (green) and after (red) the bath application of Pax (Ctrl: 13.64 ± 1.62; Pax: 13.45 ± 1.66; Z = -0.09, p = 0.96, n = 11 neurons from 6 animals).

(**G**) Ten $\Delta F/F_0$ responses evoked by single pulse stimuli at the isolated releasing synapses before (green) and after (red) the bath application of Pax.

(**H**) 10-90% rise times (Ctrl: 0.21 ± 0.03 s; Pax: 0.26 ± 0.02 s; Z = -0.53, p = 0.64, n = 11 neurons from 6 animals) and decay time constants (Ctrl: 6.42 ± 1.11; Pax: 20.02 ± 3.33; Z = 2.93, p < 0.001, n = 11 neurons from 6 animals) of $\Delta F/F_0$ responses before (green) and after (red) the bath application of Pax.

(**I**) Amplitude histograms of $\Delta F/F_0$ responses before (green) and after (red) the bath application of Pax.

(**J-K**) Average (Ctrl: 1.34 ± 0.08; Pax: 1.24 ± 0.04; Z = -1.16, p = 0.28, n = 11 neurons from 6 animals) and maximal (Ctrl: 2.27 ± 0.19; Pax: 1.91 ± 0.16; Z = -1.41, p = 0.25, n = 11 neurons from 6 animals) vesicular quanta, quantal size (Ctrl: 0.41 ± 0.06%; Pax: 0.41 ± 0.06%; Z = -0.45, p = 0.7, n =11 neurons from 6 animals), and release probability (Ctrl: 83.52 ± 3.25%; Pax: 81.50 ± 3.02%; Z = -0.36, p = 0.77, n =11 neurons from 6 animals). Asterisks indicate $p < 0.05$ (Wilcoxon tests).

Figure S5

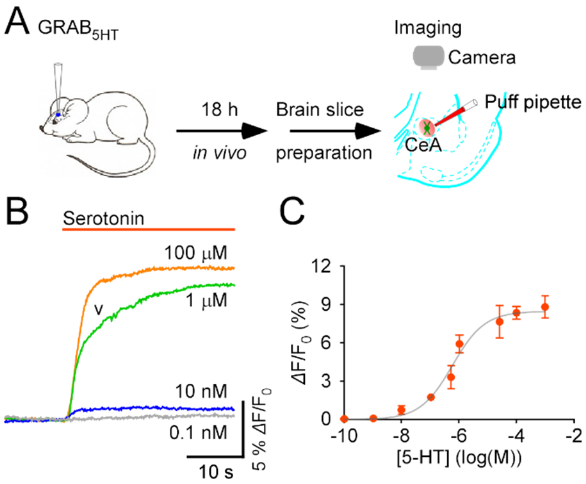

Figure S5. Calibration of $GRAB_{5HT}$ fluorescence responses to serotonin perfusion.

(**A**) Schematic of $GRAB_{5HT}$ expression and fluorescence response calibration of neurons in an *ex vivo* mouse amygdalar slice preparation.

(**B**) Steady-state fluorescence responses following the perfusion of serotonin at varying concentrations.

(**C**) Plot of fluorescence responses as a function of serotonin concentrations, fitted with a sigmoidal function $f_{(x)} = 8.52 / (1 + e^{-(\log(x)+6.23)/0.56})$ (n = 10-20 neurons from 5-8 animals, $r^2 = 0.98$, $F = 201.67$, $p < 0.0001$), with an EC_{50} of ~0.59 μM.

Figure S6

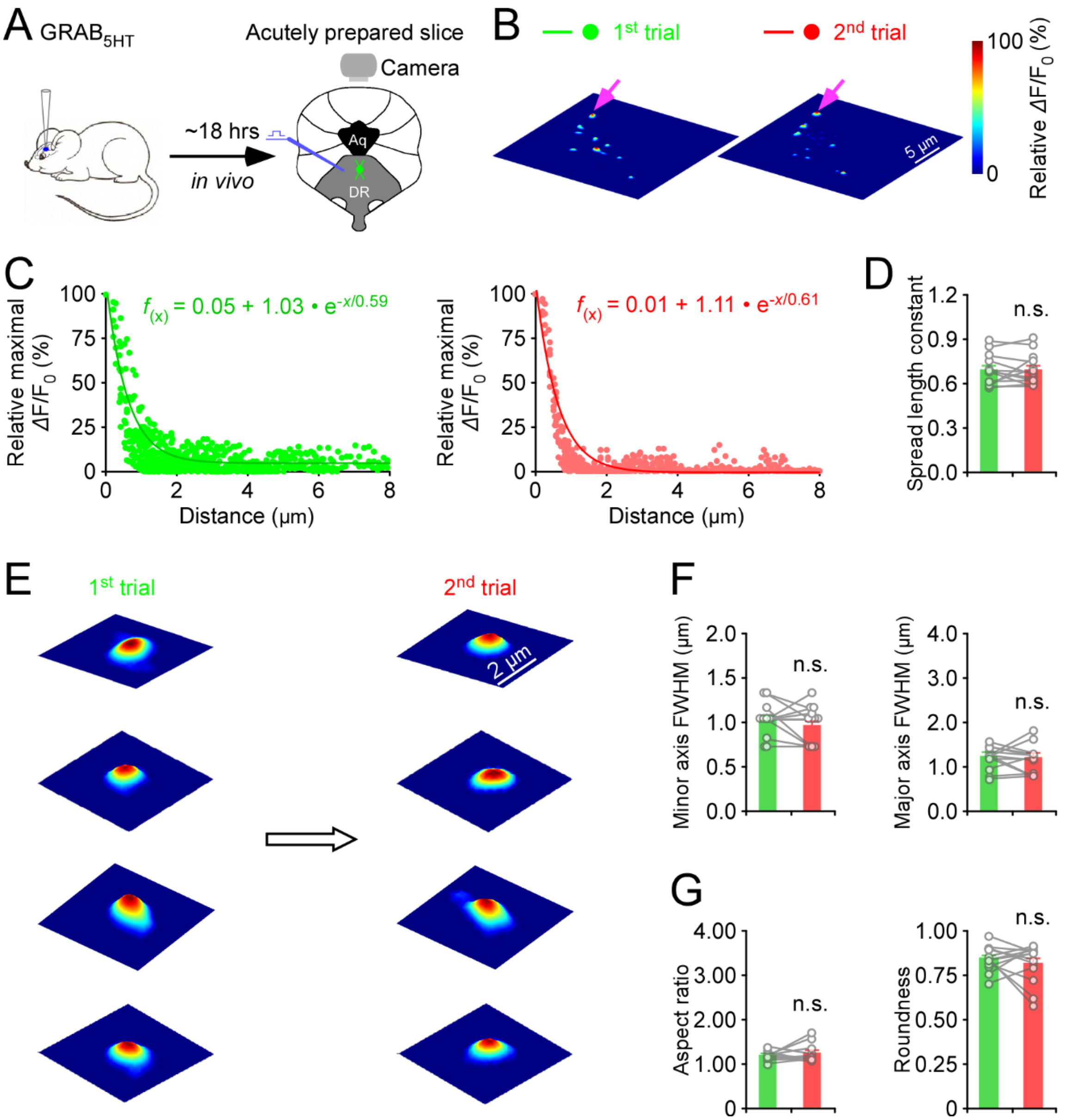

Figure S6. Serotonin spatial diffusion profile upholds during repetitive stimulation.

(**A**) Schematic of imaging experiment design in a mouse dorsal raphe nucleus (**DR**) slice preparation.

(**B**) 3D spatiotemporal profiling of electrically evoked fluorescence $\Delta F/F_0$ responses in the amygdala bathed in the normal bath solution imaged in two trials.

(**C**) Pixel-wise maximal $\Delta F/F_0$ plots at the same isolated releasing synapse indicated by the pink arrows in **B** for the two trials.

(**D**) Averaged spatial spread length constants for the two trials (1st trial: 0.69 ± 0.03 μm; 2nd trial: 0.69 ± 0.03 μm, Z = -0.25, p = 0.84, n = 13 releasing synapses from 5 animals).

(**E**) 3D profiling of $\Delta F/F_0$ responses at isolated releasing synapses for the two trials. Scale bar applied to all in **E**.

(**F**) Minor (Ctrl: 1.02 ± 0.05 μm; Ctrl: 0.96 ± 0.06 μm; Z = -1.19, p = 0.25, n = 13 releasing synapses from 5 animals) and major (Ctrl: 1.23 ± 0.07 μm; Ctrl: 1.20 ± 0.09 μm; Z = -0.59, p = 0.59, n = 13 releasing synapses from 5 animals) full width at half maximums (**FWHMs**) of serotonin diffusion profiles at isolated releasing synapses for the two trials.

(**G**) Aspect ratio (Ctrl: 1.19 ± 0.03; Ctrl: 1.25 ± 0.06; Z = 0.59, p = 0.59, n = 13 releasing synapses from 5 animals) and roundness (Ctrl: 0.83 ± 0.02; Ctrl: 0.81 ± 0.03; Z = -0.73, p = 0.50 n = 13 releasing synapses from 5 animals) values of serotonin diffusion profiles at isolated releasing synapses for the two trials.

Figure S7

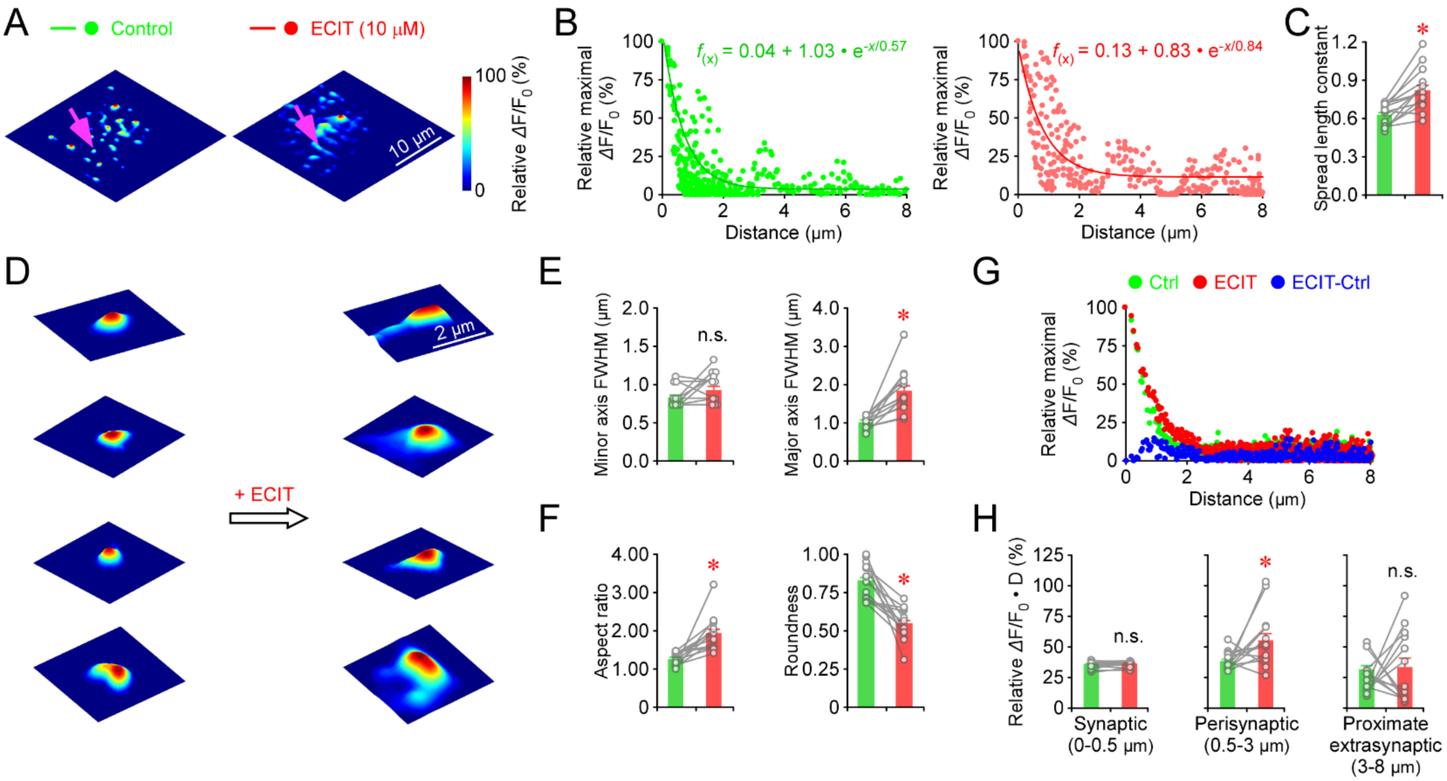

Figure S7. Transporter inhibition drives extrasynaptic diffusion of serotonin in the amygdala.

(**A**) 3D spatiotemporal profiling of electrically evoked fluorescence $\Delta F/F_0$ responses in the amygdala before (left) and after (right) the bath application of 10 μm escitalopram (ECIT).

(**B**) Pixel-wise maximal $\Delta F/F_0$ plots before (left) and after (right) the bath application of ECIT at the same isolated releasing synapse indicated by the pink arrows in **A**.

(**C**) Averaged spatial spread length constants (Ctrl: 0.62 ± 0.02 μm; ECIT: 0.82 ± 0.05 μm, Z = 3.11, p < 0.001, n = 13 releasing synapses from 5 animals).

(**D**) 3D profiling of $\Delta F/F_0$ responses at isolated releasing synapses before and after the bath application of ECIT. Note serotonin leaking out of synapses via 1−3 outlets after the bath application of ECIT (1.61 ± 0.24; n = 13 releasing synapses from 5 animals). Scale bar applied to all in **D**.

(**E**) Minor (Ctrl: 0.82 ± 0.04 μm; ECIT: 0.93 ± 0.06 μm; Z = 1.69, p = 0.11, n = 13 releasing synapses from 5 animals) and major (Ctrl: 1.01 ± 0.02 μm; ECIT: 1.82 ± 0.16 μm; Z = 3.18, p < 0.001, n = 13 releasing synapses from 5 animals) full width at half maximums (**FWHMs**) of serotonin diffusion profiles at isolated releasing synapses before and after the bath application of ECIT.

(**F**) Aspect ratio (Ctrl: 1.24 ± 0.05; ECIT: 1.92 ± 0.05; $Z = 3.18$, $p < 0.001$, $n = 13$ releasing synapses from 5 animals) and roundness (Ctrl: 0.82 ± 0.03; ECIT: 0.54 ± 0.03; $Z = -3.18$, $p < 0.001$, $n = 13$ releasing synapses from 5 animals) values of serotonin diffusion profiles at isolated releasing synapses before and after the bath application of ECIT.

(**G**) Plots of pixel-wise maximal $\Delta F/F_0$ before (green) and after (red) the bath application of ECIT and their difference (cyan; ECIT-Ctrl).

(**H**) Relative integration values of $\Delta F/F_0$ at distance of 0.5 μm (Ctrl: 0.36 ± 0.008; ECIT: 0.36 ± 0.007; $Z = 0.45$, $p = 0.68$, $n = 13$ releasing synapses from 5 animals), 0.5−3 μm (Ctrl: 0.38 ± 0.02; ECIT: 0.55 ± 0.06; $Z = 2.13$, $p = 0.03$, $n = 13$ releasing synapses from 5 animals), and 3−8 μm (Ctrl: 0.25 ± 0.04; ECIT: 0.33 ± 0.08; $Z = 0.66$, $p = 0.54$, $n = 13$ releasing synapses from 5 animals). Asterisks indicate $p < 0.05$ (Wilcoxon tests).

Figure S8

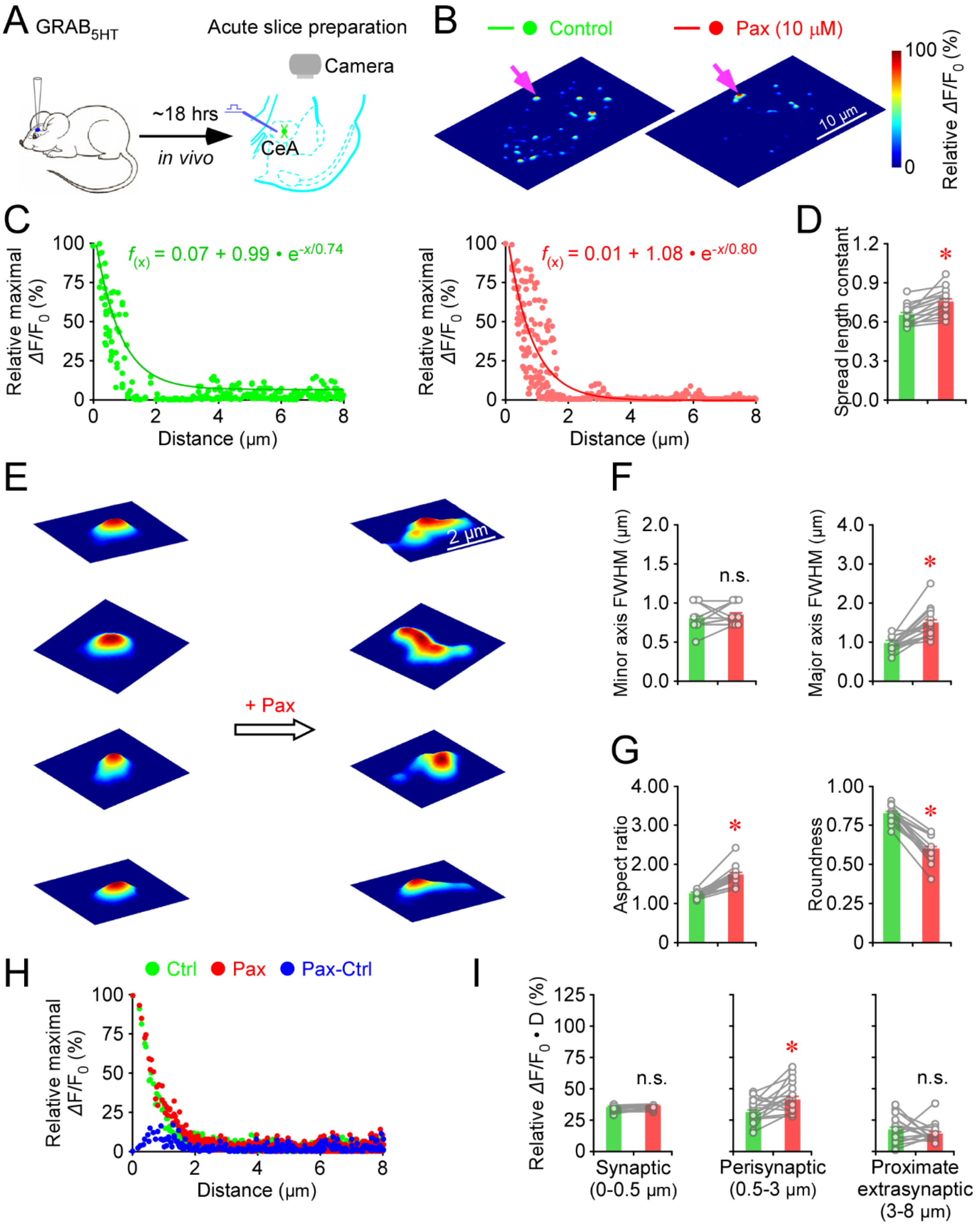

Figure S8. Transporter inhibitor paroxetine drives extrasynaptic diffusion of serotonin in the amygdala.

(**A**) Schematic of experiment design in a mouse amygdalar slice preparation. CeA: the central nucleus of the amygdala.

(**B**) 3D spatiotemporal profiling of electrically evoked fluorescence $\Delta F/F_0$ responses in the amygdala before (left) and after (right) the bath application of 10 μm paroxetine (**Pax**).

(**C**) Pixel-wise maximal $\Delta F/F_0$ plots before (left) and after (right) the bath application of Pax at the same isolated releasing synapse indicated by the pink arrows in **B**.

(**D**) Averaged spatial spread length constants (Ctrl: 0.65 ± 0.02 μm; Pax: 0.75 ± 0.02 μm, $Z = 3.52$, $p < 0.001$, $n = 16$ releasing synapses from 6 animals).

(**E**) 3D profiling of $\Delta F/F_0$ responses at isolated releasing synapses before and after the bath application of Pax. Note serotonin leaking out of synapses via 1−3 outlets after the bath application of Pax (1.56 ± 0.24; $n = 16$ releasing synapses from 6 animals). Scale bar applied to all in **E**.

(**F**) Minor (Ctrl: 0.79 ± 0.03 μm; Pax: 0.85 ± 0.03 μm; $Z = 1.28$, $p = 0.22$, $n = 16$ releasing synapses from 6 animals) and major (Ctrl: 0.97 ± 0.04 μm; Pax: 1.48 ± 0.09 μm; $Z = 3.46$, $p < 0.001$, $n = 16$ releasing synapses from 6 animals) full width at half maximums (**FWHMs**) of serotonin diffusion profiles at isolated releasing synapses before and after the bath application of Pax.

(**G**) Aspect ratio (Ctrl: 1.22 ± 0.02; Pax: 1.73 ± 0.06; $Z = 3.52$, $p < 0.001$, $n = 16$ releasing synapses from 6 animals) and roundness (Ctrl: 0.82 ± 0.01; Pax: 0.58 ± 0.02; $Z = -3.52$, $p < 0.001$, $n = 16$ releasing synapses from 6 animals) values of serotonin diffusion profiles at isolated releasing synapses before and after the bath application of Pax.

(**H**) Plots of pixel-wise maximal $\Delta F/F_0$ before (green) and after (red) the bath application of Pax and their difference (cyan; ECIT-Ctrl).

(**I**) Relative integration values of $\Delta F/F_0$ at distance of 0.5 μm (Ctrl: 0.35 ± 0.007; Pax: 0.36 ± 0.004; $Z = 1.40$, $p = 0.18$, $n = 16$ releasing synapses from 6 animals), 0.5−3 μm (Ctrl: 0.32 ± 0.02; Pax: 0.43 ± 0.03; $Z = 2.59$, $p = 0.008$, $n = 16$ releasing synapses from 6 animals), and 3−8 μm (Ctrl: 0.17 ± 0.03; Pax: 0.14 ± 0.02; $Z = -0.88$, $p = 0.40$, $n = 16$ releasing synapses from 6 animals). Asterisks indicate $p < 0.05$ (Wilcoxon tests).

Figure S9

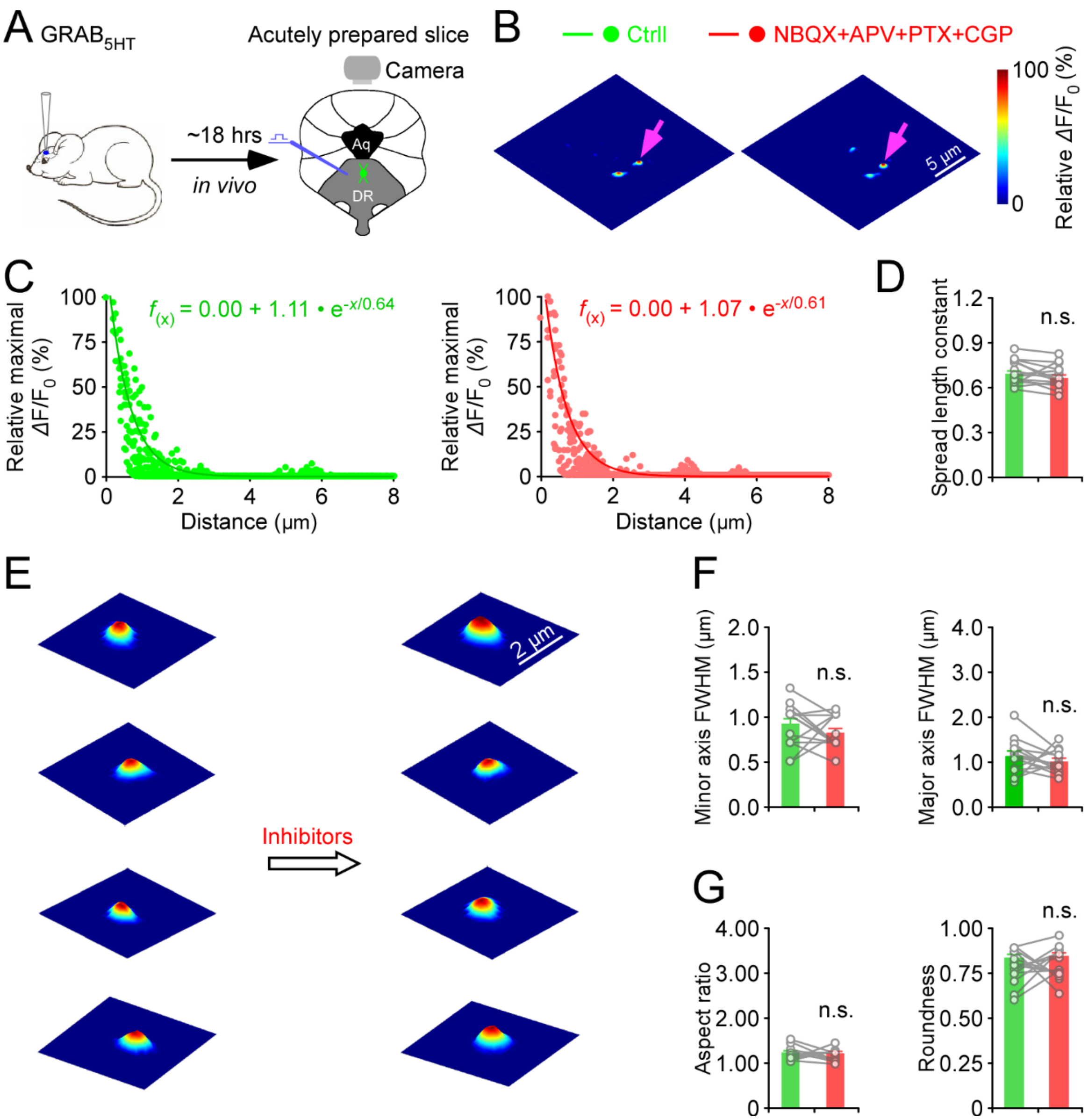

Figure S9. Serotonin diffusion profile is independent of glutamatergic and GABAergic transmission.

(**A**) Schematic of experiment design in a mouse dorsal raphe nucleus (**DR**) slice preparation.

(**B**) 3D spatiotemporal profiling of electrically evoked fluorescence $\Delta F/F_0$ responses in the amygdala before (left) and after (right) the bath application of 5 μM NBQX, 100 μM AP5, 50 μM PTX, and 5 μM CGP. Scale bar applied to all in **B**.

(**C**) Pixel-wise maximal $\Delta F/F_0$ plots at the same isolated releasing synapses indicated by the pink arrows in **B** before (left) and after (right) the bath application of inhibitors NBQX, AP5, PTX, and CGP.

(**D**) Averaged spatial spread length constants (Ctrl: 0.69 ± 0.02 μm; Drug: 0.66 ± 0.02 μm, *Z* = -1.76, *p* = 0.08, *n* = 15 releasing synapses from 5 animals) before (left) and after (right) the bath application of inhibitors NBQX, AP5, PTX, and CGP.

(**E**) 3D profiling of $\Delta F/F_0$ responses at isolated releasing synapses before (left) and after (right) the bath application of inhibitors NBQX, AP5, PTX, and CGP. Scale bar applied to all in **E**.

(**F**) Minor (Ctrl: 0.92 ± 0.06 μm; Drug: 0.82 ± 0.04 μm; *Z* = -1.30, *p* = 0.21, *n* = 15 releasing synapses from 5 animals) and major (Ctrl: 1.13 ± 0.1 μm; Drug: 1.00 ± 0.06 μm; *Z* = -1.42, *p* = 0.17, *n* = 15 releasing synapses from 5 animals) full width at half maximums (**FWHMs**) of serotonin diffusion profiles at isolated releasing synapses before (left) and after (right) the bath application of inhibitors NBQX, AP5, PTX, and CGP.

(**G**) Aspect ratio (Ctrl: 1.22 ± 0.04; Drug: 1.21 ± 0.03; *Z* = -0.11, *p* = 0.93, *n* = 15 releasing synapses from 5 animals) and roundness (Ctrl: 0.83 ± 0.02; Drug: 0.84 ± 0.02; *Z* = 0.28, *p* = 0.80 *n* = 15 releasing synapses from 5 animals) values of serotonin diffusion profiles at isolated releasing synapses before (left) and after (right) the bath application of inhibitors NBQX, AP5, PTX, and CGP

Figure S10

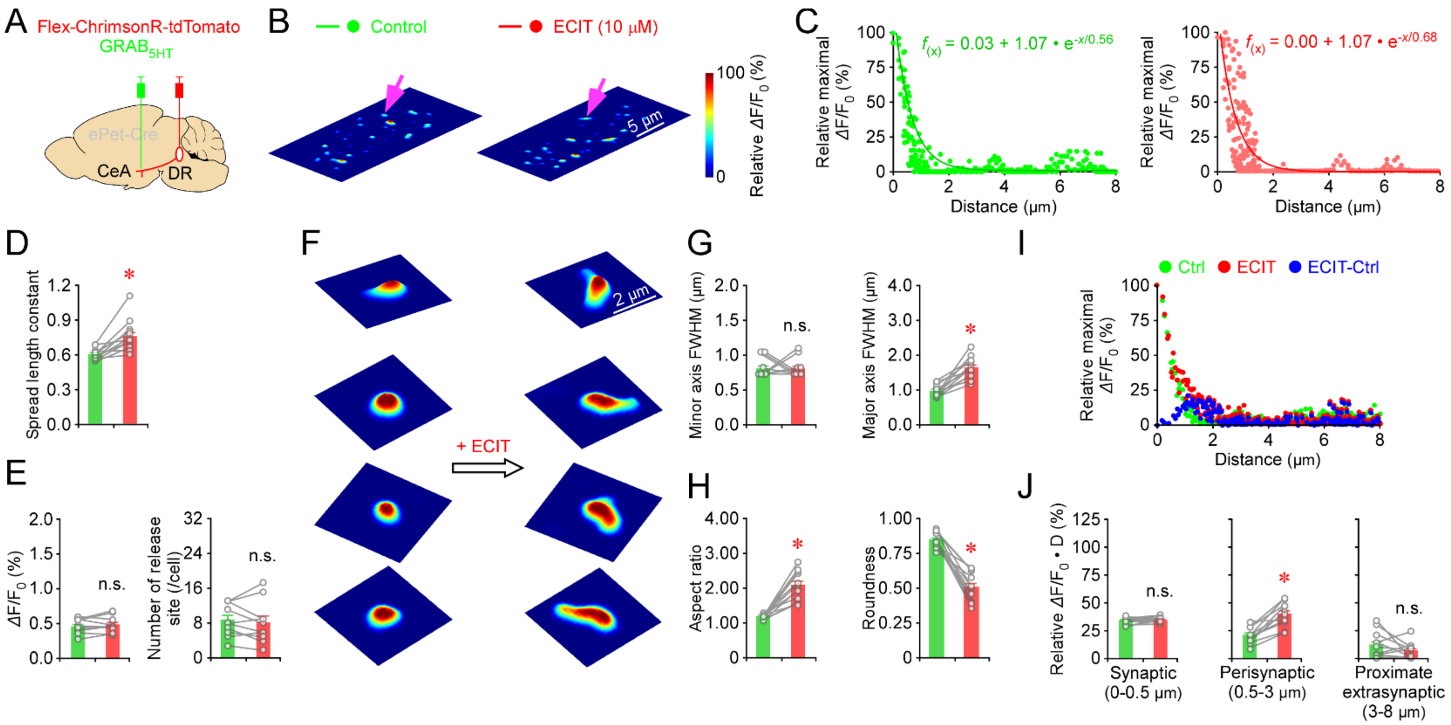

Figure S10. Transporter inhibition drives extrasynaptic diffusion of serotonin released optogenetically.

(**A**) Schematic of experimental design in the ePet-Cre transgenic mice with AAV-Syn-FLEX-ChrimsonR-tdTomato expressed in the dorsal raphe nucleus (**DR**) and $GRAB_{5HT}$ in the central nucleus of the amygdala (**CeA**).

(**B**) 3D spatiotemporal profiling of optogenetically evoked fluorescence $\Delta F/F_0$ responses in the amygdala before (left) and after (right) the bath application of 10 μm escitalopram (**ECIT**). Scale bar applied to all in **B**.

(**C**) Pixel-wise maximal $\Delta F/F_0$ plots before (left) and after (right) the bath application of ECIT at the same isolated releasing synapse indicated by the pink arrows in **B**.

(**D**) Averaged spatial spread length constants (Ctrl: 0.60 ± 0.01 μm; ECIT: 0.76 ± 0.04 μm, Z = 3.06, p < 0.001, n = 11 neurons from 5 animals).

(**E**) Peak amplitudes (Ctrl: 0.46 ± 0.03%; ECIT: 0.49 ± 0.03%, Z = 0.97, p = 0.38, n = 10 neurons from 5 animals) and releasing synapse counts before (green) and after (red) the bath application of ECIT (Ctrl: 8.70 ± 1.15; ECIT: 8.10 ± 1.49; Z = -0.77, p = 0.49, n = 10 neurons from 5 animals).

(**F**) 3D profiling of $\Delta F/F_0$ responses at isolated releasing synapses before and after the bath application of ECIT. Note serotonin leaking out of synapses via 1−3 outlets after the bath application of ECIT (1.58 ± 0.19; *n* = 11 releasing synapses from 5 animals). Scale bar applied to all in **F**.

(**G**) Minor (Ctrl: 0.81 ± 0.04 μm; ECIT: 0.80 ± 0.04 μm; $Z = -0.77$, $p = 0.49$, $n = 11$ releasing synapses from 5 animals) and major (Ctrl: 0.97 ± 0.05 μm; ECIT: 1.63 ± 0.09 μm; $Z = 3.06$, $p < 0.001$, $n = 11$ releasing synapses from 5 animals) full width at half maximums (**FWHMs**) of serotonin diffusion profiles at isolated releasing synapses before and after the bath application of ECIT.

(**H**) Aspect ratio (Ctrl: 1.18 ± 0.02; ECIT: 2.06 ± 0.11; $Z = 3.06$, $p < 0.001$, $n = 11$ releasing synapses from 5 animals) and roundness (Ctrl: 0.85 ± 0.02; ECIT: 0.50 ± 0.03; $Z = -3.06$, $p < 0.001$, $n = 11$ releasing synapses from 5 animals) values of serotonin diffusion profiles at isolated releasing synapses before and after the bath application of ECIT.

(**I**) Plots of pixel-wise maximal $\Delta F/F_0$ before (green) and after (red) the bath application of ECIT and their difference (cyan; ECIT-Ctrl).

(**J**) Relative integration values of $\Delta F/F_0$ at distance of 0.5 μm (Ctrl: 0.34 ± 0.009; ECIT: 0.34 ± 0.007; $Z = -0.078$, $p = 0.97$, $n = 11$ releasing synapses from 5 animals), 0.5−3 μm (Ctrl: 0.21 ± 0.02; ECIT: 0.40 ± 0.03; $Z = 2.20$, $p = 0.03$, $n = 11$ releasing synapses from 5 animals), and 3−8 μm (Ctrl: 0.13 ± 0.04; ECIT: 0.07 ± 0.02; $Z = -1.41$, $p = 0.17$, $n = 11$ releasing synapses from 5 animals). Asterisks indicate $p < 0.05$ (Wilcoxon tests).

Figure S11

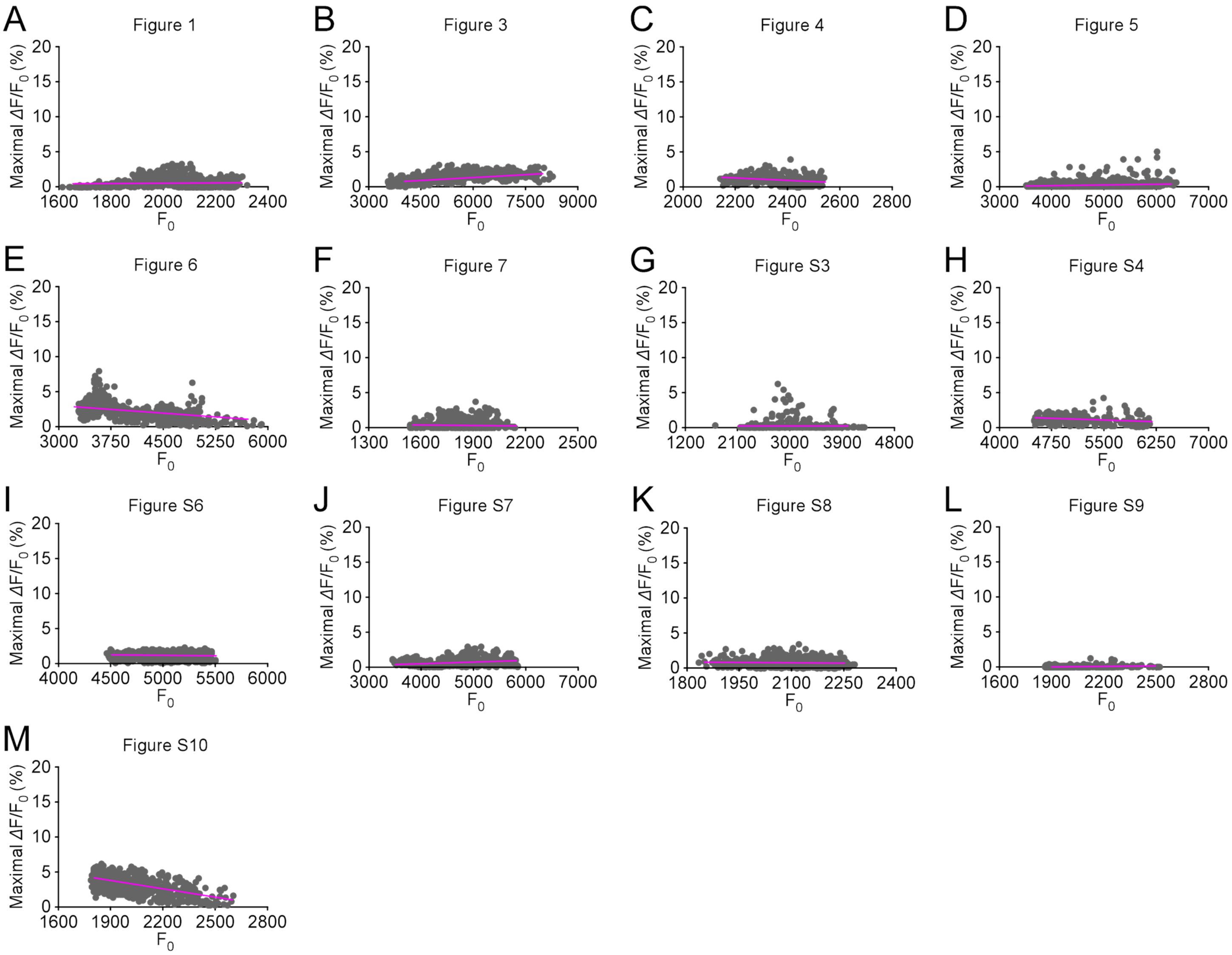

Figure S11. Fluorescence responses are largely independent of $GRAB_{5HT}$ expression levels.

(**A**) Plots of $\Delta F/F_0$ against F_0 of the $GRAB_{5HT}$ expressing neuron in DR, shown in Figure 1 (Slop of regression line = 0.0002; Normality test $p \leqslant 0.001$; Constant variance test $p \leqslant 0.001$; $r^2 = 0.0029$; $F = 19.46$, $n = 6{,}653$; $p < 0.001$).

(**B**) Plots of $\Delta F/F_0$ against F_0 of the $GRAB_{5HT}$ expressing neuron in DR, shown in Figure 3 for control (Slop of regression line = 0.00029; Normality test $p \leqslant 0.001$; Constant variance test $p \leqslant 0.001$; $r^2 = 0.33$; $F = 4605.06$, $n = 9{,}207$; $p < 0.001$).

(**C**) Plots of $\Delta F/F_0$ against F_0 of the $GRAB_{5HT}$ expressing neuron in CeA, shown in Figure 4 (Slop of regression line = - 0.0017; Normality test $p \leqslant 0.001$; Constant variance test $p = 0.0021$; $r^2 = 0.070$; $F = 264.13$, $n = 3{,}518$; $p < 0.001$).

(**D**) Plots of $\Delta F/F_0$ against F_0 of the $GRAB_{5HT}$ expressing neuron in CeA, shown in Figure 5 (Slop of regression line = 0.00008; Normality test $p \leqslant 0.001$; Constant variance test $p \leqslant 0.001$; $r^2 = 0.026$; $F = 380.03$, $n = 14{,}220$; $p < 0.001$).

(**E**) Plots of $\Delta F/F_0$ against F_0 of the $GRAB_{5HT}$ expressing neuron in DR, shown in Figure 6 for control (Slop of regression line = -0.0006; Normality test $p \leqslant 0.001$; Constant variance test $p \leqslant 0.001$; $r^2 = 0.20$; $F = 2266.71$, $n = 9{,}347$; $p < 0.001$).

(**F**) Plots of $\Delta F/F_0$ against F_0 of the $GRAB_{5HT}$ expressing neuron in DR, shown in Figure 7 for control (Slop of regression line = -0.00039; Normality test $p \leqslant 0.001$; Constant variance test $p \leqslant 0.001$; $r^2 = 0.046$; $F = 505.80$, $n = 10{,}524$; $p < 0.001$).

(**G**) Plots of $\Delta F/F_0$ against F_0 of the $GRAB_{5HT}$ expressing neuron in CeA, shown in Figure S3 for control (Slop of regression line = 0.000024; Normality test $p \leqslant 0.001$; Constant variance test $p \leqslant 0.001$; $r^2 = 0.0003$; $F = 1.18$, $n = 3{,}589$; $p = 0.27$).

(**H**) Plots of $\Delta F/F_0$ against F_0 of the $GRAB_{5HT}$ expressing neuron in CeA, shown in Figure S4 for control (Slop of regression line = - 0.00029; Normality test $p \leqslant 0.001$; Constant variance test $p \leq 0.001$; $r^2 = 0.057$; $F = 167.72$, $n = 2{,}763$; $p < 0.001$).

(**I**) Plots of $\Delta F/F_0$ against F_0 of the $GRAB_{5HT}$ expressing neuron in DR, shown in Figure S6 (Slop of regression line = - 0.00016; Normality test $p \leqslant 0.001$; Constant variance test $p \leqslant 0.001$; $r^2 = 0.0072$; $F = 34.56$, $n = 4{,}736$; $p < 0.001$).

(**J**) Plots of $\Delta F/F_0$ against F_0 of the $GRAB_{5HT}$ expressing neuron in CeA, shown in Figure S7 (Slop of regression line = 0.00025; Normality test $p \leqslant 0.001$; Constant variance test $p \leqslant 0.001$; $r^2 = 0.063$; $F = 293.13$, $n = 4{,}395$; $p < 0.001$).

(**K**) Plots of $\Delta F/F_0$ against F_0 of the $GRAB_{5HT}$ expressing neuron in CeA, shown in Figure S8 for control (Slop of regression line = - 0.00047; Normality test $p \leqslant 0.001$; Constant variance test $p \leqslant 0.001$; $r^2 = 0.0045$; $F = 33.85$, $n = 6{,}716$; $p < 0.001$).

(**L**) Plots of $\Delta F/F_0$ against F_0 of the $GRAB_{5HT}$ expressing neuron in DR, shown in Figure S9 for control (Slop of regression line = 0.00016; Normality test $p \leqslant 0.001$; Constant variance test $p \leqslant 0.001$; $r^2 = 0.050$; $F = 292.39$, $n = 5595$; $p < 0.001$).

(**M**) Plots of $\Delta F/F_0$ against F_0 of the $GRAB_{5HT}$ expressing neuron in CeA, shown in Figure S10 for control (Slop of regression line = - 0.004; Normality test $p \leqslant 0.001$; Constant variance test $p \leqslant 0.001$; $r^2 = 0.37$; $F = 4324.90$, $n = 7{,}384$; $p < 0.001$).

A representative subset of 10% of the data points is displayed for clarity.

Movie S1. ECIT elongates $\Delta F/F_0$ responses at a $GRAB_{5HT}$ expressing neuron.

(Please click the start button in **B** to view the video clips)

A

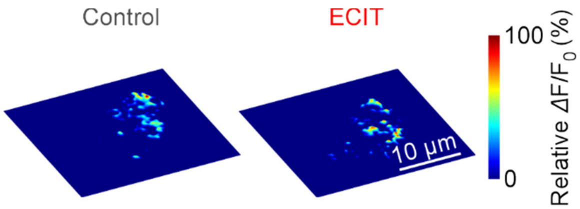

B

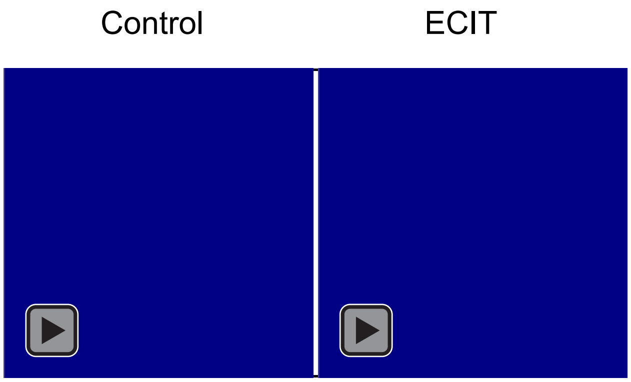

Movie S2. ECIT elongates $\Delta F/F_0$ responses at a single serotonin release site.

(Please click the start button in **B** to view the video clips)

A

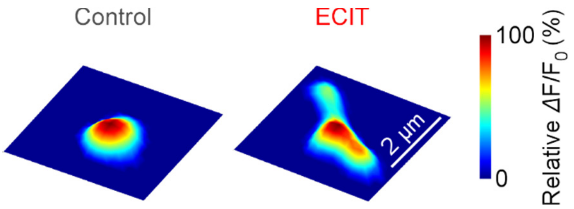

B

Control ECIT

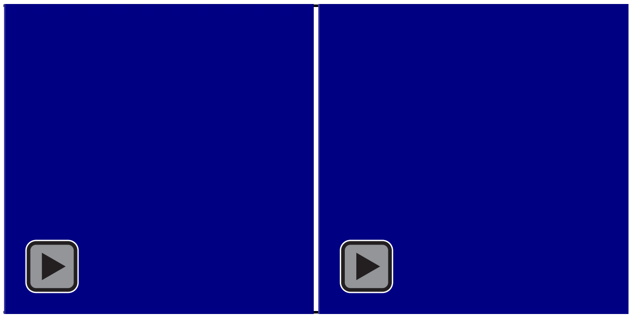

Movie S3. ECIT elongates optogenetically evoked $\Delta F/F_0$ responses at a single serotonin release site.

(Please click the start button in **B** to view the video clips)

A

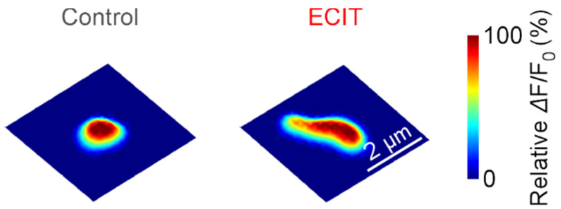

B

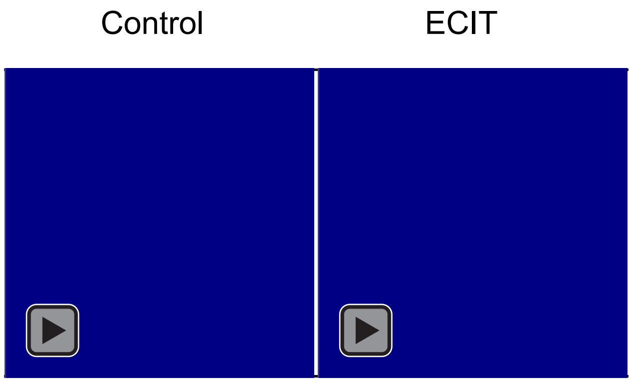

Movie S4. Frequency-dependent $\Delta F/F_0$ responses.

(Please click the start button in **B** to view the video clips)

A

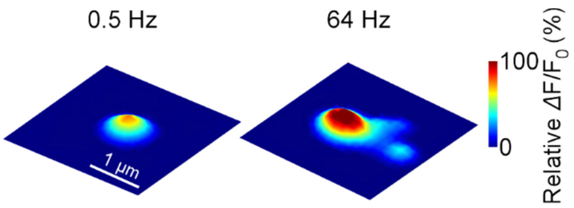

B

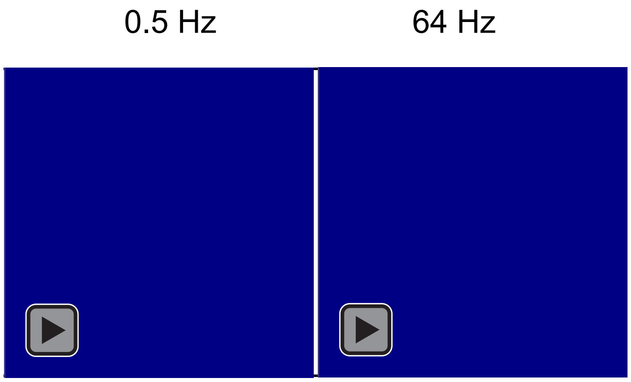

Movie S5. Elevated $\Delta F/F_0$ responses in response to presumably increased recruitment of axons

(Please click the start button in **B** to view the video clips)

A

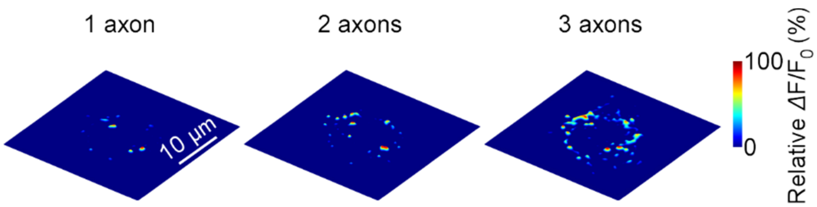

B

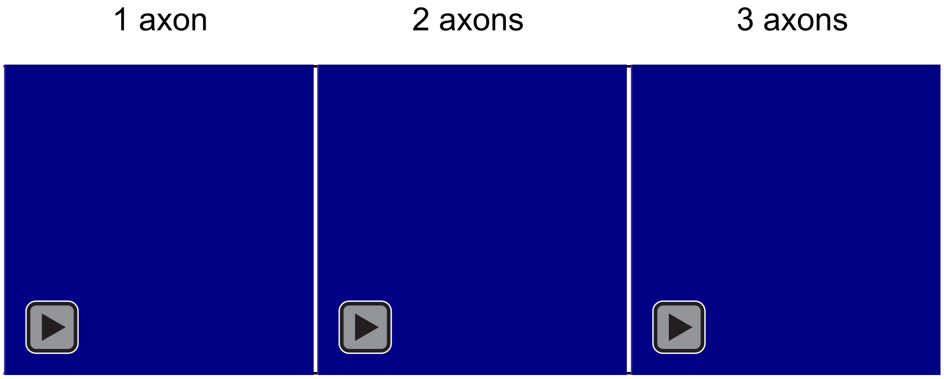

Supplement: Supplementary file 1 — Supplemental materials [file 41380_2025_2930_MOESM1_ESM.pdf]
